# PHOTOLYASE/BLUE LIGHT RECEPTOR2 regulates chrysanthemum flowering by compensating for gibberellin perception

**DOI:** 10.1093/plphys/kiad503

**Published:** 2023-09-19

**Authors:** Xin Zhao, Wenwen Liu, Palinuer Aiwaili, Han Zhang, Yanjie Xu, Zhaoyu Gu, Junping Gao, Bo Hong

**Affiliations:** Beijing Key Laboratory of Development and Quality Control of Ornamental Crops, Department of Ornamental Horticulture, China Agricultural University, Beijing 100193, China; State Key Laboratory of Plant Physiology and Biochemistry, College of Biological Sciences, China Agricultural University, Beijing 100193, China; Beijing Key Laboratory of Development and Quality Control of Ornamental Crops, Department of Ornamental Horticulture, China Agricultural University, Beijing 100193, China; Beijing Key Laboratory of Development and Quality Control of Ornamental Crops, Department of Ornamental Horticulture, China Agricultural University, Beijing 100193, China; Beijing Key Laboratory of Development and Quality Control of Ornamental Crops, Department of Ornamental Horticulture, China Agricultural University, Beijing 100193, China; Beijing Key Laboratory of Development and Quality Control of Ornamental Crops, Department of Ornamental Horticulture, China Agricultural University, Beijing 100193, China; Beijing Key Laboratory of Development and Quality Control of Ornamental Crops, Department of Ornamental Horticulture, China Agricultural University, Beijing 100193, China; Beijing Key Laboratory of Development and Quality Control of Ornamental Crops, Department of Ornamental Horticulture, China Agricultural University, Beijing 100193, China; Beijing Key Laboratory of Development and Quality Control of Ornamental Crops, Department of Ornamental Horticulture, China Agricultural University, Beijing 100193, China

## Abstract

The gibberellins (GAs) receptor GA INSENSITIVE DWARF1 (GID1) plays a central role in GA signal perception and transduction. The typical photoperiodic plant chrysanthemum (*Chrysanthemum morifolium*) only flowers when grown in short-day photoperiods. In addition, chrysanthemum flowering is also controlled by the aging pathway, but whether and how GAs participate in photoperiod- and age-dependent regulation of flowering remain unknown. Here, we demonstrate that photoperiod affects *CmGID1B* expression in response to GAs and developmental age. Moreover, we identified PHOTOLYASE/BLUE LIGHT RECEPTOR2, an atypical photocleavage synthase, as a CRYPTOCHROME-INTERACTING bHLH1 interactor with which it forms a complex in response to short days to activate *CmGID1B* transcription. Knocking down *CmGID1B* raised endogenous bioactive GA contents and GA signal perception, in turn modulating the expression of the aging-related genes *MicroRNA156* and *SQUAMOSA PROMOTER BINDING PROTEIN-LIKE3*. We propose that exposure to short days accelerates the juvenile-to-adult transition by increasing endogenous GA contents and response to GAs, leading to entry into floral transformation.

## Teaser

Short days accelerate the juvenile-to-adult transition by increasing endogenous gibberellin biosynthesis and perception in chrysanthemum, leading to entry into floral transformation.

## Introduction

Flowering is a critical developmental transition in the plant life cycle, as flowering at the correct time is essential for plant reproductive success and survival. When to flower is determined by a complex regulatory network integrating environmental cues and endogenous signals (Amasino and Michaels 2010; Wahl et al. 2013). Angiosperms have evolved diverse strategies to coordinately regulate floral transition based on environmental stimuli, such as photoperiod and vernalization and endogenous cues, such as the gibberellin (GA) and the aging pathways (Srikanth and Schmid 2011; Song et al. 2013).

Photoperiod is arguably one of the most pervasive environmental factors affecting plant growth and development (Sullivan and Deng 2003). Plants are classified into 3 major types based on their flowering response to changes in daylength (photoperiod): long day (LD), short day (SD), and day neutral (Srikanth and Schmid 2011). The photoperiodic pathway begins with the perception of light signals by photoreceptors in leaves. Among these photoreceptors, cryptochromes (CRYs) are conserved photolyase-related blue light receptors that mediate light responses in plants and animals. There are 3 CRYs in Arabidopsis (*Arabidopsis thaliana*), CRY1, CRY2, and CRY3, of which CRY1 and CRY2 are involved in regulating flowering via stabilizing CONSTANS (CO), a core component of the photoperiod pathway (Schepens et al. 2004; Du et al. 2020). Several members of the basic helix-loop-helix (bHLH) family of transcription factors appear to be particularly important, as several directly bind to the E-box elements (CANNTG) in the *FLOWERING LOCUS T* (*FT*) promoter to activate *FT* transcription, namely, CRYPTOCHROME-INTERACTING bHLH1 (CIB1), CIB2, CIB4, and CIB5 (Liu et al. 2008; Luo et al. 2021; Zhou et al. 2021). Previous studies have demonstrated that CIB1, CRY2, and CO form a protein complex in response to blue light, whereby the CRY2-CO and CRY2-CIB1 modules interact to regulate *FT* transcription and flowering (Liu et al. 2018). However, the relationship between CIB1 and other flowering pathways is largely unknown. In addition, the atypical PHOTOLYASE/BLUE LIGHT RECEPTOR2 (PHR2) was also annotated in Arabidopsis, but little is known about its possible functions (Ahmad and Jarillo 1998).

The phytohormone GA is widely involved in the flowering decision (Kumar et al. 2012; Yan et al. 2020). The initiation of GA signaling involves 4 components: bioactive GAs, the GA-receptor GA INSENSITIVE DWARF1 (GID1), the central repressor DELLA, and the specific F-box protein SLEEPY1 (SLY1), which degrades DELLA (Murase et al. 2008; Nemoto et al. 2017). The GA-receptor GID1 was identified in rice (*Oryza sativa*), and putatively orthologous genes have been described in a wide range of angiosperms since (Ueguchi-Tanaka et al. 2005; Hirano et al. 2007). Three homologous *GID1* genes, *GID1A*, *GID1B*, and *GID1C*, have been identified in Arabidopsis (Nakajima et al. 2006). GID1s bind active GAs, interact with DELLAs, and induce their degradation through the 26S proteasome pathway mediated by the Skp-Cullin-F-box complex SCF^SLY1^. Most reports on crosstalk between the GA pathway and other pathways in the context of flowering have mainly focused on the interaction between DELLA and other key regulatory factors (Griffiths et al. 2006; Wu and Poethig 2006; de Lucas et al. 2008; Wang et al. 2009; Wang 2014; Wang et al. 2016; Xu et al. 2016). For example, DELLAs directly bind to CO, a core regulator of the photoperiodic pathway, and inhibit the interaction of CO with NF-YB2, leading to inhibition of the transcriptional ability of CO and downregulation of *FT* expression under LD in Arabidopsis (Wang et al. 2016; Xu et al. 2016). DELLAs also interact with another key *FT* activator, PHYTOCHROME INTERACTING FACTOR4 (PIF4) and repress its DNA-binding ability, thereby inhibiting PIF4-activated gene expression (de Lucas et al. 2008). Nevertheless, much less is known about whether and how GID1s regulate flowering in response to changes in photoperiod.

The aging pathway is dominated by endogenous developmental cues that prevent flowering at the juvenile stage and ensure flowering at the adult stage even in the absence of positive external environmental cues (Wang 2014). MicroRNA156 (miR156) and its target gene *SQUAMOSA PROMOTER BINDING PROTEIN-LIKE* (*SPL*) are critical components of the aging pathway and regulate the transition from juvenile to adult phases and flowering (Wu and Poethig 2006; Wang et al. 2009). Over the course of seedling growth and plant development, the abundance of miR156 gradually decreases, leading to an increase in *SPL* transcript levels and the induction of the downstream flowering integrator genes *SUPPRESSOR OF OVEREXPRESSION OF CO1* (*SOC1*), *APETALA1* (*AP1*), and *LEAFY* (*LFY*) in the apical meristem (Wu and Poethig 2006; Wang 2014). The role for miR156 and *SPL*s in flowering is conserved in many angiosperms, such as maize (*Zea mays*) (Chuck et al. 2011), rice (Xie et al. 2006), potato (*Solanum tuberosum*) (Bhogale et al. 2014), and chrysanthemum (*Chrysanthemum morifolium*) (Wei et al. 2017). We previously showed that Nuclear Factor subunit YB8 (CmNF-YB8) is a key factor sensing aging signals that directly trigger the miR156-SPL module and regulate flowering time in chrysanthemum (Wei et al. 2017). However, it is largely unknown whether the aging pathway mediated by miR156-SPL is involved in regulating flowering in response to SD conditions and endogenous GA phytohormone levels.

Chrysanthemum is a typical SD herbaceous perennial species whose floral transition is closely related to the induction of exogenous environmental and endogenous phytohormones and developmental signals. Hence, chrysanthemum can be used as a useful model to study the role of daylength in the transition from vegetative growth to inflorescence meristem identity. Studies of flowering in chrysanthemum have described the role of the zinc finger protein B-box 24 (BBX24) and antiflorigenic FT/TFL1 family protein as repressors of flowering under LD conditions (Higuchi et al. 2013; Higuchi and Hisamatsu 2015). However, few studies have looked at the interplay between photoperiod, endogenous phytohormones, and development. In this study, we report that the GA-receptor CmGID1B plays an important role in integrating photoperiod information from the CmCIB1-PHR2 complex and plant age from the cmo-miR156-CmSPL3 module to coordinate endogenous GA levels and signal perception. We show that CmGID1B accelerates the juvenile-to-adult transition when chrysanthemum plants are transferred to SD conditions.

## Results

### GA biosynthesis and signal perception are induced in SD conditions

**Figure 1.**
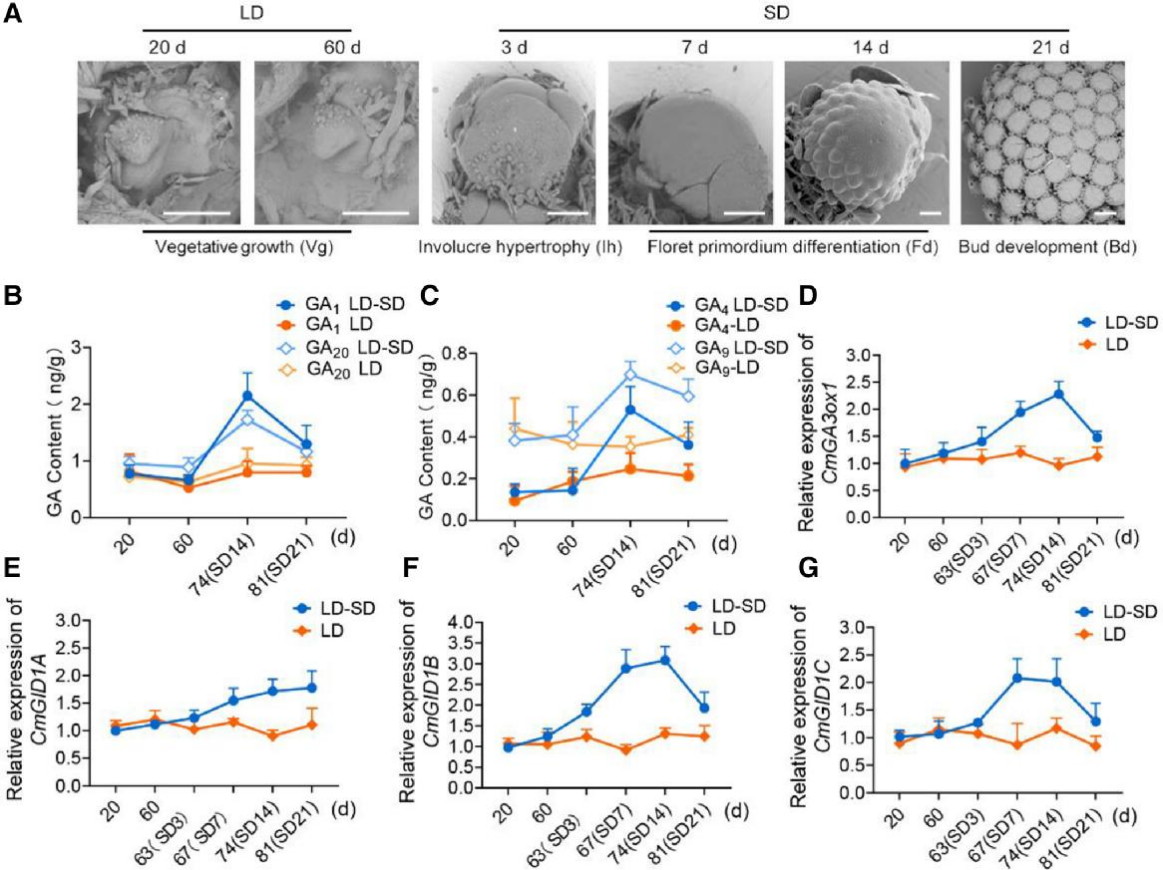
GA and *CmGID1* expression levels rise in SDs in chrysanthemum. **A)** Morphology of shoot apices in the transition period from vegetative growth to flowering in chrysanthemum. After 60 d of growth in LDs, chrysanthemum plants were transferred to SDs, and the shoot apices and inflorescence were dissected and observed by scanning electron microscopy. Scale bars, 100 *μ*m. **B** and **C)**. Contents of active GA_1_ and its precursor GA_20_, and active GA_4_ and its precursor GA_9_ as analyzed by LC-MS/MS in chrysanthemum shoot apices in the transition from vegetative growth to flowering. **D** to **G)** Relative expression levels of *CmGA3ox1* and *CmGID1A, B, C* analyzed by RT-qPCR in chrysanthemum in the transition from vegetative growth to flowering. *UBIQUITIN* was used as an internal control. The results are the means of 3 biological replicates with standard.

To explore the effect of a SD photoperiod on flowering in chrysanthemum, we inspected the morphology of shoot apices grown in LD conditions and transferred to SD conditions (Fig. 1A). Under LD conditions, plants remained in the vegetative growth stage. The apical meristem of plants moved to SDs for 3 d began to hypertrophy and formed the involucral primordium after 7 d in SDs. Floret primordium differentiation and the development of floret primordia occurred after 14 and 21 d, respectively, in SDs.

To investigate whether GAs are involved in the floral transition induced by SDs, we measured endogenous GA levels of chrysanthemum plants grown in LD conditions and after the switch to SDs. We determined that the abundance of bioactive GA_1_ and its precursor GA_20_ increases substantially after transfer to SDs and rose 2.2- and 1.8-fold relative to LD levels, respectively, when plants entered the floret primordium differentiation stage (Fig. 1B). Similarly, the concentrations of bioactive GA_4_ and its precursor GA_9_ increased by 2.5- and 2.0-fold, respectively, relative to LD levels (Fig. 1C). We also evaluated the expression of the GA biosynthesis genes *GA_3_ oxidase 1* (*CmGA3ox1*) and *CmGA20ox1* under both LD and SD conditions. *CmGA3ox1* expression continuously increased in SDs from 3 to 14 d (Fig. 1D), as did *CmGA20ox1* expression, although not to the same extent (Supplemental Fig. S1A).

In addition, we confirmed the relationship between GAs and SD photoperiod during chrysanthemum flowering by applying exogenous GA or the GA biosynthetic inhibitor paclobutrazol (PAC). Under SD conditions, the time to flowering was significantly delayed in plants treated with 100 *μ*m PAC compared with controls (Supplemental Fig. S1B to D). Conversely, under LD conditions, flowering was accelerated after application of 100 *μ*m GA_4/7_. These results indicate that the effects of SDs on flowering time are associated with GA levels in chrysanthemum.

To investigate how SD conditions induce flowering in response to GAs, we identified 3 genes encoding the GA receptor in chrysanthemum, namely, *CmGID1A*, *CmGID1B*, and *CmGID1C*, and established that all CmGID1 proteins are located in the nucleus and cytoplasm (Supplemental Fig. S2). Similar to the changes in GA contents and *CmGA3ox1* expression reported above, the expression of *CmGID1*s increased substantially upon transfer from LDs to SDs (Fig. 1E to G). *CmGID1B* expression levels increased by up to 3.2-fold over LD levels, more than *CmGID1A* or *CmGID1C* (which increased by 1.7- and 2.3-fold, respectively). *CmGID1B* and *CmGID1C* followed a diurnal rhythm under SD conditions (Supplemental Fig. S2). These data suggest that *CmGID1*s might participate in the SD induction of floral transition through the GA pathway in chrysanthemum.

### CmGID1s regulate flowering

**Figure 2.**
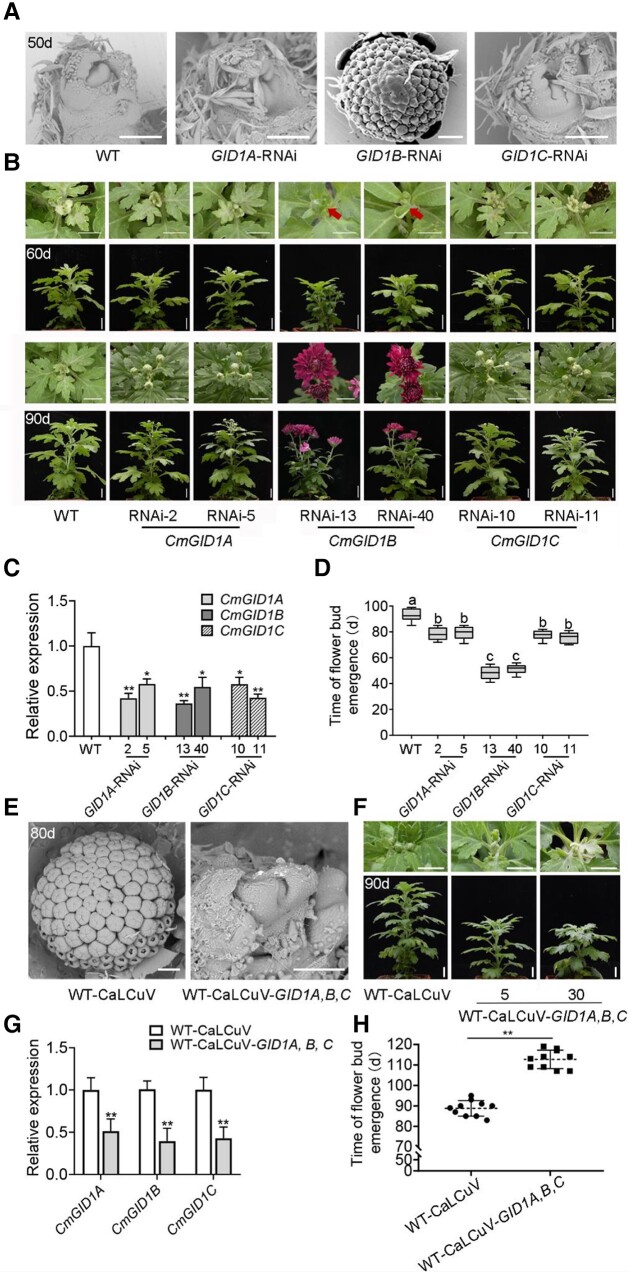
CmGID1s are involved in the regulation of flowering in chrysanthemum. **A)** Shoot apices of WT and *CmGID1-*RNAi plants at 50 d under LD observed by scanning electron microscopy. Scale bars, 100 *μ*m. **B)** Representative phenotypes of WT and *CmGID1-*RNAi plants at 60 d under LD conditions and at 90 d under SD conditions, including 60 d of LD conditions and 30 d of SD conditions. Scale bars, 1 cm. **C)** Relative expression levels of *CmGID1A, B, C* analyzed by RT-qPCR in WT and *CmGID1-*RNAi plants grown for 30 d under LD conditions. **D)** Time of flower bud emergence of WT and *CmGID1-*RNAi plants. Eighteen samples were used to calculate the days of flower bud emergence; *n* = 18. Center line, median; box limits, upper and lower quartiles; whiskers, 1.5× interquartile range; points, outliers. Different lowercase letters indicate significant differences according to Duncan's multiple range test (*P* < 0.05). **E)** Shoot apices of WT plants (50-d-old) infected with CaLCuV or CaLCuV-amiR-CmGID1A, B, C after 20 d under LD conditions and 10 d under SD conditions observed by scanning electron microscopy. Scale bars, 100 *μ*m. **F)** Representative phenotypes of WT plants (50-d-old) infected with CaLCuV or CaLCuV-amiR-CmGID1A, B, C after 20 d under LD conditions and 20 d under SD conditions. Scale bars, 1 cm. **G)** Relative expression levels of *CmGID1A, B, C* analyzed by RT-qPCR in WT plants (50-d-old) infected with CaLCuV or CaLCuV-amiR-CmGID1A, B, C after 5 d under LD conditions. **H)** Time of flower bud emergence of WT plants (50-d-old) infected with CaLCuV or CaLCuV-amiR-CmGID1A, B, C. The results are the means of 3 biological replicates with SD. Asterisks indicate significant differences according to a Student's *t*-test in **C**), **G**), **H**) (*P < 0.05, **P < 0.01).

To investigate whether CmGID1s influence the floral transition and transformation, we specifically knocked down the transcript levels of *CmGID1A*, *CmGID1B*, or *CmGID1C* in chrysanthemum by generating the corresponding *CmGID1*-RNA interference (RNAi) lines (Fig. 2C). We determined that *CmGID1B*-RNAi plants can complete floral transformation without SD induction. Microscopy observations showed that the apical meristem of *CmGID1B*-RNAi plants enters the early stage of floret primordium differentiation after spending 50 d in LD conditions, in contrast to wild-type (WT), *CmGID1A*-RNAi, and *CmGID1C*-RNAi plants that remain in the vegetative stage (Fig. 2, A and B).

As we kept plants under LD conditions for 60 d, we observed flower bud emergence in *CmGID1B*-RNAi lines (Fig. 2B). To ensure that all plants have the ability to receive the necessary signals for floral induction, we transferred all plants to SD conditions for further cultivation for 30 d. Flower buds of *CmGID1B*-RNAi lines were bloomed, and *CmGID1A*-RNAi and *CmGID1C*-RNAi plants entered the flower bud development stage, while WT plants were still at the vegetative stage (Fig. 2, B and D).

In order to clarify why the function of *CmGID1s* is inconsistent with previous reports and explain the real function of *CmGID1s* in flowering transition of chrysanthemums, we simultaneously silenced all 3 *CmGID1s* in the WT plants (Fig. 2G). We observed that compared with WT-CaLCuV plants, silencing *CmGID1A, B, C* significantly delayed the flowering time. The apical meristem of WT-CaLCuV plants had completed floret primordium differentiation on day 80 through microscopic observation, and plants had visible flower buds on day 90, in contrast to CaLCuV-amiR-CmGID1A, B, C plants that still remained in the vegetative stage (Fig. 2, E, F, and H). We also transiently overexpressed *CmGID1B* in chrysanthemums and found that *CmGID1B*-overexpressed plants showed an early flowering phenotype (Supplemental Fig. S3, A to C). Taken together, we conclude that CmGID1s can regulate the chrysanthemum flowering and CmGID1B plays a more major role.

### CmGID1B regulates the juvenile-to-adult transition through the aging pathway

**Figure 3.**
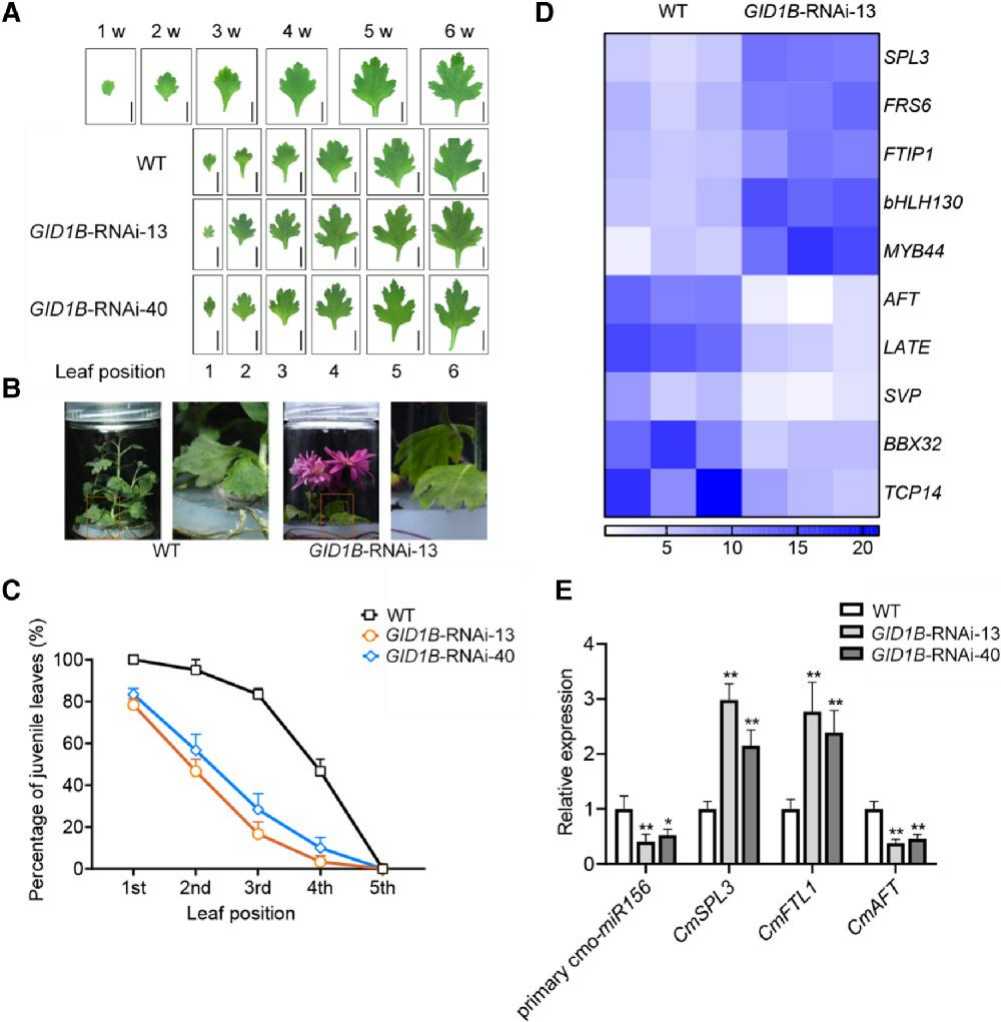
*CmGID1B-*RNAi plants undergo an accelerated juvenile vegetative phase. **A)** Morphology of leaves of WT and *CmGID1B-*RNAi chrysanthemum plants grown under LD conditions for 40 d. The first row shows the leaf morphology at 1 to 6 wk of normal growth. Juvenile leaves are small with no, or minimal, marginal serrations. Samples were photographed at the same time, and images were digitally extracted for comparison. Scale bars, 1 cm. **B)** Morphology of leaves of WT and *CmGID1B-*RNAi chrysanthemum plants grown under LD conditions for 90 d. The parts in the box are partially enlarged for observation. **C)** Percentage of juvenile leaves among the first 5 leaves in WT and *CmGID1B-*RNAi chrysanthemum plants. **D)** Heatmap showing the expression of DEGs in WT and *CmGID1B-*RNAi plants. Dark blue represents high expression levels. **E)** Relative expression levels of abundance of the age-related genes primary *cmo-miR156*, *CmSPL3*, *CmFTL1*, and *CmAFT* analyzed by RT-qPCR in WT and *CmGID1B-*RNAi plants grown for 40 d under LD conditions. The results are the means of 3 biological replicates with SD. Asterisks indicate significant differences according to a Student's *t*-test in **E**) (*P < 0.05, **P < 0.01).

The morphology of juvenile leaves is usually used as an indicator of the juvenile-to-adult transition in chrysanthemum. To investigate why the flowering time of *CmGID1B*-RNAi lines was advanced, we inspected leaf morphology and scored the proportion of juvenile leaves from *CmGID1B*-RNAi lines and the WT. All first-emerging leaves, 95% of second leaves, and 83% of third leaves from WT plants were juvenile, in that they were small and had no, or minimal, marginal serration. By contrast, more than 17%, 44%, and 72% of first-, second-, and third-emerging leaves, respectively, of *CmGID1B*-RNAi lines had an adult morphology, as they were larger and had serrated margins (Fig. 3, A to C).

To ascertain that the influence of *CmGID1B* on flowering is exerted through the aging pathway in chrysanthemum, we identified differentially expressed genes (DEGs) between the leaves of the WT and *CmGID1B*-RNAi line 13 by transcriptome deep sequencing (RNA-seq). We obtained 3,393 DEGs in *CmGID1B*-RNAi plants, of which 2,155 were upregulated and 1,238 were downregulated relative to the WT (Supplemental Data Set 1). We focused on DEGs annotated as components of the aging or flowering pathways: *CmSPL3*, *FT-INTERACTING PROTEIN1* (*FTIP1*), and *CmAFT*. *CmSPL3* and *CmFTIP1* expression was higher in *CmGID1B*-RNAi plants than in the WT, while that of *CmAFT* was lower (Fig. 3D). To validate the RNA-seq results, we evaluated the expression of *CmSPL3* and *CmAFT* in *CmGID1B*-RNAi plants by reverse transcription quantitative PCR (RT-qPCR). The expression levels of *CmSPL3* and *CmAFT* were consistent with those from the RNA-seq data (Fig. 3E). We also observed that the primary transcript of *cmo*-*miR156* is significantly downregulated, while *CmFT-like1* (*CmFTL1*) expression was significantly upregulated in *CmGID1B*-RNAi plants compared with the WT (Fig. 3E). In addition, we also found significant differences in the transcriptional abundance of *CmbHLH130*, *CmMYB44*, *SHORT VEGETATIVE PHASE*, *B-BOX DOMAIN 32*, and *TEOSINTE BRANCHED CYCLOIDEA AND PCF 14* in the transcriptome data, suggesting that they may function as downstream regulatory genes of GA signal. These results suggest that knocking down *CmGID1B* accelerated the juvenile-to-adult transition by regulating the expression of aging pathway genes in chrysanthemum.

### CmGID1B regulates GA biosynthesis and perception in chrysanthemum

**Figure 4.**
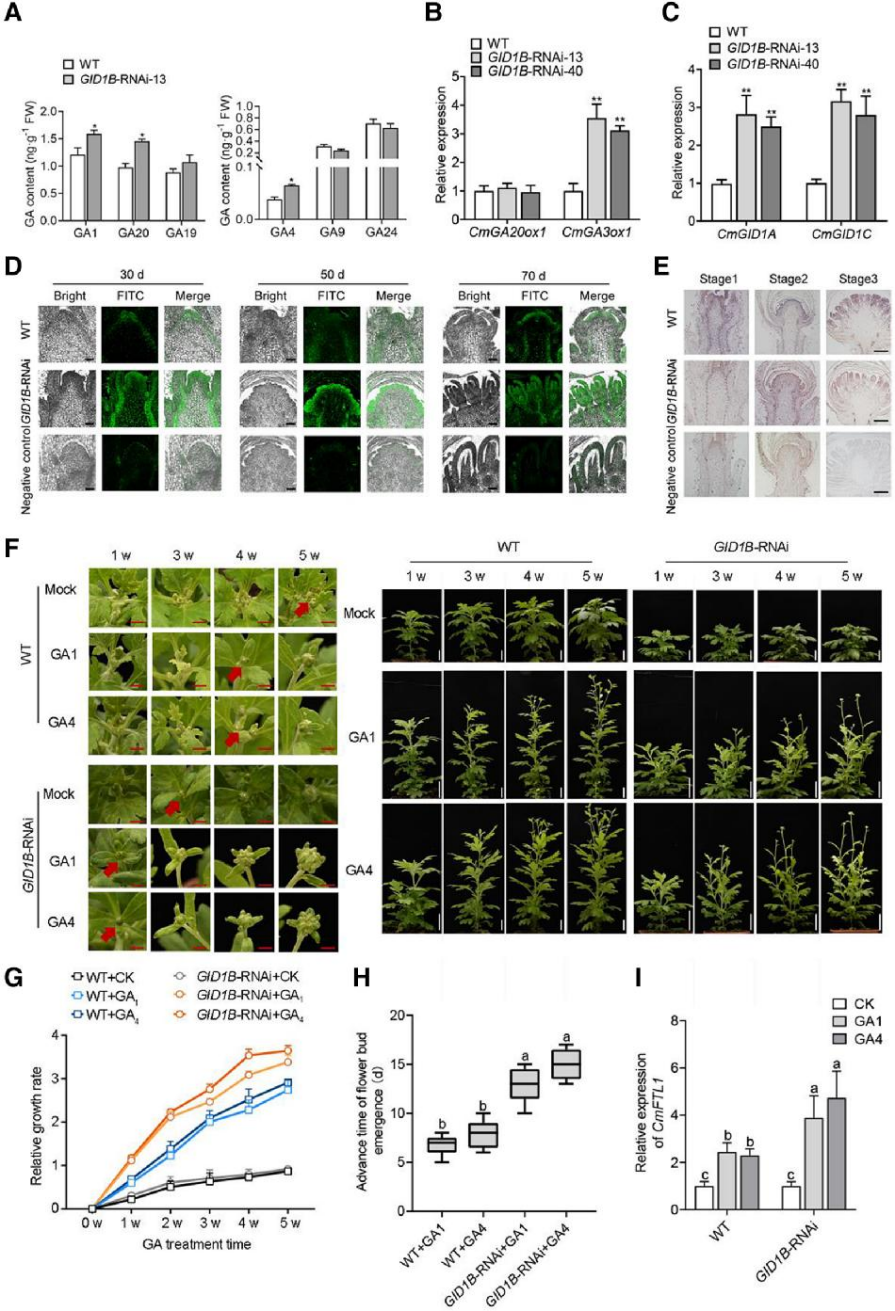
Higher bioactive GAs contents and perception in *CmGID1B-*RNAi plants. **A)** GA contents in the shoot apices of WT and *CmGID1B-*RNAi plants grown under LD conditions for 40 d. **B)** Relative expression of the GA biosynthesis genes *CmGA20ox1* and *CmGA3ox1* analyzed by RT-qPCR in WT and *CmGID1B-*RNAi plants. **C)** Relative expression of *CmGID1A* and *CmGID1C* analyzed by RT-qPCR in WT and *CmGID1B-*RNAi plants. The results are the means of 3 biological replicates with SD. Asterisks indicate significant differences according to a Student's *t*-test in **A** to **C)** (*P < 0.05, **P < 0.01). **D)** Immunolocalization of GA in the shoot apices of WT and *CmGID1B-*RNAi plants at different stages of floral transformation, using a polyclonal anti-GA antibody. Green fluorescence is the immune signal. For the negative controls, the antibody was omitted. Scale bars, 100 *μ*m. **E)***In situ* hybridization analysis of *CmGID1B* transcript abundance and localization (shown in purple) in apical meristems of WT and *CmGID1B-*RNAi plants at different stages of floral transformation. The negative control was hybridized with the sense probe. Scale bars, 200 *μ*m. **F)** Representative phenotypes of WT and *CmGID1B-*RNAi plants after treatment with GA_1_ or GA_4_. Red arrows indicate flower bud emergence. Scale bars (left images), 0.5 cm. Scale bars (right images), 1 cm. **G)** Relative growth rates of WT and *CmGID1B-*RNAi plants after GA treatment. **H)** Acceleration of flower bud emergence of WT and *CmGID1B-*RNAi plants after GA treatment. The average bud days of WT and *CmGID1B-*RNAi plants after spraying GA and CK were recorded, and the D-value was calculated. Twelve samples were used to calculate the advance days of flower bud emergence; *n* = 12. Center line, median; box limits, upper and lower quartiles; whiskers, 1.5× interquartile range; points, outliers. **I)** Relative *CmFTL1* expression level analyzed by RT-qPCR in WT and *CmGID1B-*RNAi plants after GA treatment. The results are the means of 3 biological replicates with SD. Different lowercase letters indicate significant differences according to Duncan's multiple range test in **H)** and **I)** (*P* < 0.05).

To clarify the cause of the shorter juvenile period and early flowering seen following *CmGID1B* knockdown, we measured endogenous GA contents in *CmGID1B*-RNAi and WT plants. We determined that bioactive GA_1_ and GA_4_ levels are significantly higher in *CmGID1B*-RNAi plants than in WT plants (Fig. 4A). We also evaluated the expression of the GA biosynthesis genes *CmGA20ox1* and *CmGA3ox1*. *CmGA3ox1* expression was significantly upregulated, while *CmGA20ox1* expression was comparable between *CmGID1B*-RNAi and WT plants (Fig. 4B).

In addition, we performed immunolocalization studies with an anti-GA antibody and in situ hybridization analysis of *CmGID1B* transcript levels and distribution in apical meristems during the transformation from vegetative to floral growth. We mainly detected GAs in the apical meristem and vascular bundles at the vegetative growth stage, with a concentrated signal in involucre and floret primordia during floret primordium differentiation (Fig. 4D). The *CmGID1B* signal obtained by in situ hybridization was consistent with that of GAs (Fig. 4E). We detected a stronger immunofluorescence signal in *CmGID1B*-RNAi plants compared with in the WT, during both the vegetative growth stage and the floret primordium differentiation stage (Fig. 4D).

Based on the increase of GA contents in *CmGID1B*-RNAi plants, we detected the expression of the other 2 GID1 members. The result showed that the expression of *CmGID1A* and *CmGID1C* was significantly upregulated in *CmGID1B*-RNAi plants (Fig. 4C). In addition, the other 2 GID1 members’ expression was also increased to varying degrees in *CmGID1A*-RNAi and *CmGID1C*-RNAi plants (Supplemental Fig. S4). We next applied 100 *μ*m GA_1_ or GA_4_ onto *CmGID1B*-RNAi plants to investigate whether their response to GA had changed. Indeed, we noticed that time to flower bud emergence advances by 13 or 15 d in *CmGID1B*-RNAi plants after application of 100 *μ*m GA_1_ or GA_4_, respectively, compared with controls. The same treatment (100 *μ*m GA_1_ or GA_4_) only advanced emergence by 6 or 7 d in WT plants, respectively (Fig. 4, F and H). We also observed that relative growth rate in *CmGID1B*-RNAi plants is substantially higher than that of WT plants after application of GA_1_ or GA_4_, as measured by stem length (Fig. 4, F and G). In addition, *CmFTL1* expression was 3.8- or 4.3-fold higher in *CmGID1B*-RNAi plants after application of 100 *μ*m GA_1_ or GA_4_, respectively, compared with controls. *CmFTL1* expression only increased by 2.3- and 2.2-fold in WT plants under the same conditions (Fig. 4I). The abundance of the primary *cmo*-*miR156* transcript and *CmAFT* expression were significantly downregulated, while *CmSPL3* expression was significantly upregulated after application of 100 *μ*m GA_1_ or GA_4_ compared with controls (Supplemental Fig. S5). These results demonstrate that knocking down of *CmGID1B* increases the contents of endogenous bioactive GA_1_ and GA_4_. The increased GA contents might induce the upregulation of *CmGID1A* and *CmGID1C* expression, which in turn enhances the response to GA and then accelerates the maturation and flowering.

### CmCIB1 interacts with CmPHR2 to regulate *CmGID1B* expression

**Figure 5.**
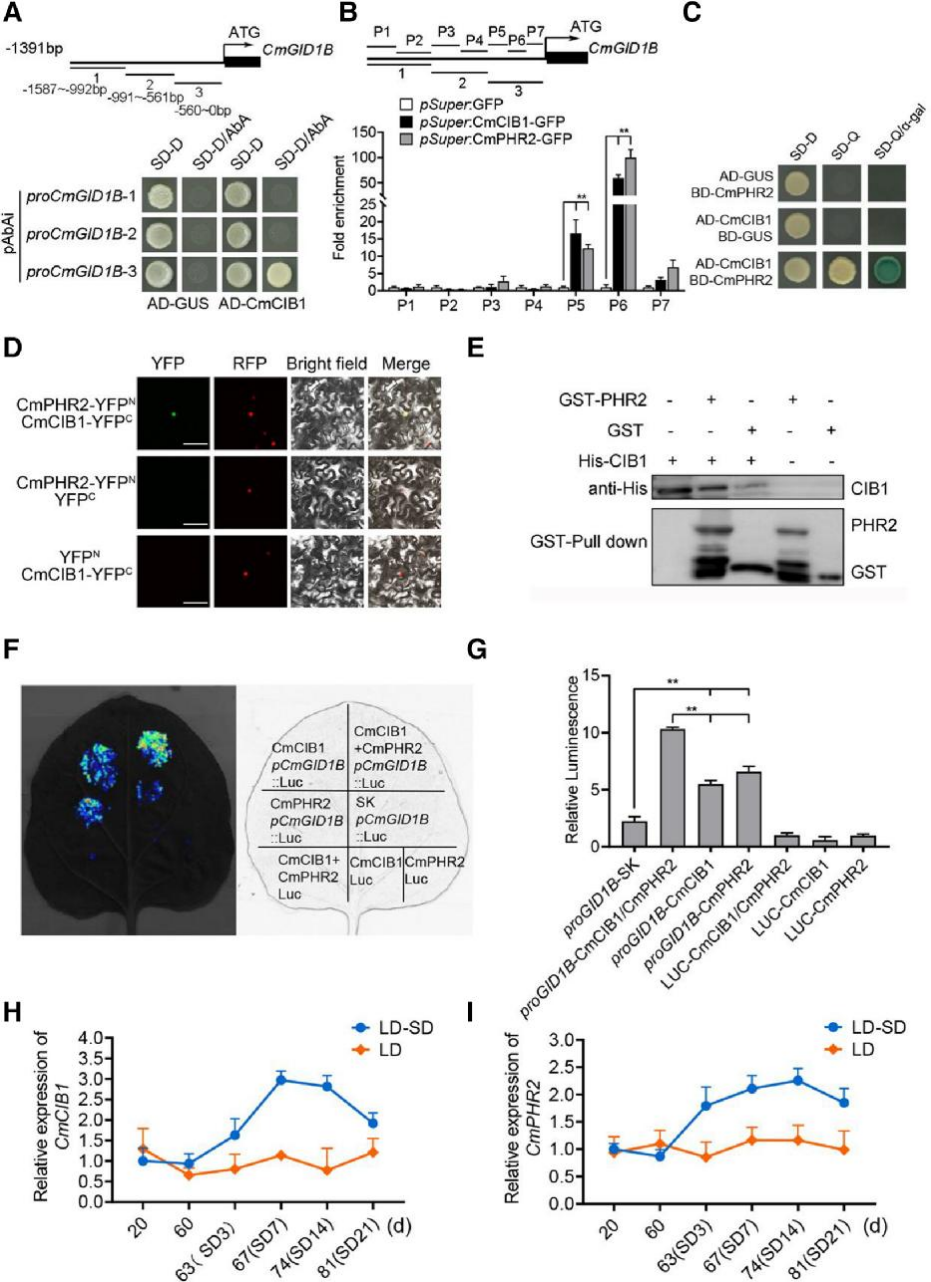
CmCIB1 interacts with CmPHR2 and directly regulates the expression of *CmGID1B*. **A)** Analysis of CmCIB1 binding to the *CmGID1B* promoter in a Y1H assay. Interaction between bait and prey constructs was determined by cell growth on synthetic defined (SD) (only used in this figure) medium lacking Leu and Ura (SD–D [double dropout]) and containing 200 mg/*µ*L AbA. **B)** ChIP-PCR of the indicated fragments (P1 to P7) of the *CmGID1B* promoter. Chromatin from *pSuper*:CmCIB1-GFP or *pSuper*:CmPHR2-GFP chrysanthemum plants was immunoprecipitated with an anti-GFP antibody. *pSuper*:*GFP* chrysanthemum plants served as a negative control. The amount of the indicated DNA fragment was determined by qPCR and normalized to the *pSuper*:GFP control (set to 1 for each fragment). **C)** Y2H assays evaluating the interaction between CmCIB1 and CmPHR2. The bait BD-CmPHR2 and prey AD-CmCIB1 plasmids were cotransformed into yeast strain Y2HGold. Transformants were grown on SD medium lacking Leu and Trp (SD-D) and then transferred to SD medium lacking Leu, Trp, and His (SD–Q [quadruple dropout]) and with X-α-gal (SD-Q+α-gal). The *GUS* sequence was inserted into pGADT7 or pGBKT7 as a negative control. **D)** Interaction of CmCIB1 and CmPHR2 in a BiFC assay. *N. benthamiana* leaves were coinfiltrated with *CmPHR2-YFP^N^* and *CmCIB1-YFP^C^* constructs and visualized by confocal microscopy 3 d after infiltration. Combinations of *CmPHR2-YFP^N^* and *YFP^C^*, and *CmCIB1-YFP^C^* and *YFP^N^* were used as negative controls. Scale bars, 100 *μ*m. **E)** GST pull-down assays showing the interaction of CmCIB1 and CmPHR2. Recombinant His-CmCIB1 was detected with anti-His antibody. GST-CmPHR2 and GST were detected with anti-GST antibody. **F)** Representative images of firefly luciferase activity showing CmCIB1 and CmPHR2 inducing transcription from the *CmGID1B* promoter. **G)** Normalized LUC activity of the indicated samples, shown as a LUC/REN ratio. The LUC/REN ratio was normalized to samples coinfiltrated with the empty reporter and 2 effectors (*LUC* + *CmCIB1-SK* + *CmPHR2-SK*), which were set to 1. **H** and **I)** Relative expression levels of *CmPHR2***H)** and *CmCIB1***I)** analyzed by RT-qPCR in chrysanthemum in the transition from vegetative growth to flowering. The results are the means of 3 biological replicates with SD. Asterisks indicate significant differences according to a Student's *t*-test in **B** and **G)** (**P < 0.01).

To explore the upstream regulators of *CmGID1B*, we performed a yeast 1-hybrid (Y1H) screen with different *CmGID1B* promoter regions using a cDNA library prepared from leaves exposed to SD. We identified the bHLH family member CmCIB1 as a potential upstream regulator. We confirmed the interaction between CmCIB1 and the *CmGID1B* promoter in a targeted Y1H assay (Fig. 5A). Chromatin immunoprecipitation (ChIP)-PCR assay also showed that CmCIB1 binds to the P5 or P6 fragment of the *CmGID1B* promoter (Fig. 5B).

To clarify how CmCIB1 regulates *CmGID1B* expression in response to photoperiod, we identified the photolyase/blue light photoreceptor CmPHR2 as a potential CIB1-interacting protein via a yeast 2-hybrid (Y2H) screen. Both CmCIB1 and CmPHR2 can be localized to the nucleus (Supplemental Fig. S6B). We confirmed that CmCIB1 can interact with CmPHR2 by targeted Y2H (Fig. 5C). We also carried out a bimolecular fluorescence complementation (BiFC) assay and observed strong signals of yellow fluorescent protein (YFP) in *Nicotiana benthamiana* leaf cells transiently coexpressing CmPHR2-YFP^N^ and CmCIB1-YFP^C^ (Fig. 5D). In contrast, we did not observe any detectable YFP signals in the negative controls, CmPHR2-YFP^N^ and YFP^C^, and YFP^N^ and CmCIB1-YFP^C^. These results indicate that CmPHR2 can interact with CmCIB1 in vivo. We then performed pull-down experiments with recombinant GST-CmPHR2 protein and GST protein as baits and His-CmCIB1 as prey. The result showed that His-CmCIB1 was pulled down by GST-CmPHR2 but not GST, indicating that CmPHR2 interacts with CmCIB1 in vitro (Fig. 5E).

Moreover, a ChIP-PCR assay using an anti-GFP antibody and chrysanthemum plants overexpressing *CmPHR2-GFP* showed that CmPHR2 is also enriched at the P5 or P6 fragment of the *CmGID1B* promoter (Fig. 5B).

We used a dual-luciferase reporter assay to evaluate the regulatory activity of the CmCIB1-PHR2 complex in the *CmGID1B* promoter in vivo. To this end, we placed the firefly luciferase (*LUC*) reporter gene under the control of the *CmGID1B* promoter (*proCmGID1B*:*LUC*) and used *35S:CmCIB1* and *35S:CmPHR2* as the effector constructs. Coinfiltration of *proCmGID1B*:*LUC* and *35S:CmCIB1* or *35S:CmPHR2* into *N. benthamiana* leaves resulted in higher relative LUC activity than when the empty vector and *proCmGID1B*:*LUC* were coinfiltrated. Coinfiltration of both effector constructs with the reporter into *N. benthamiana* leaves produced more relative LUC activity compared with either effector construct alone (Fig. 5, F and G).

We also evaluated the expression of *CmCIB1* and *CmPHR2* under LD and SD conditions. Both genes were upregulated upon transfer from LDs to SDs, while their expression levels remained relatively constant in LDs, which was reminiscent of *CmGID1B* (Figs. 1F and 5, H and I). *CmCIB1* and *CmPHR2* also appeared to follow a similar diurnal rhythm as *CmGID1B* under SDs (Supplemental Fig. S6A). These data suggest that the CmCIB1-PHR2 complex directly activates the transcription of *CmGID1B* in response to SD.

### CmCIB1-PHR2-GIDIB regulate flowering through the aging pathway

**Figure 6.**
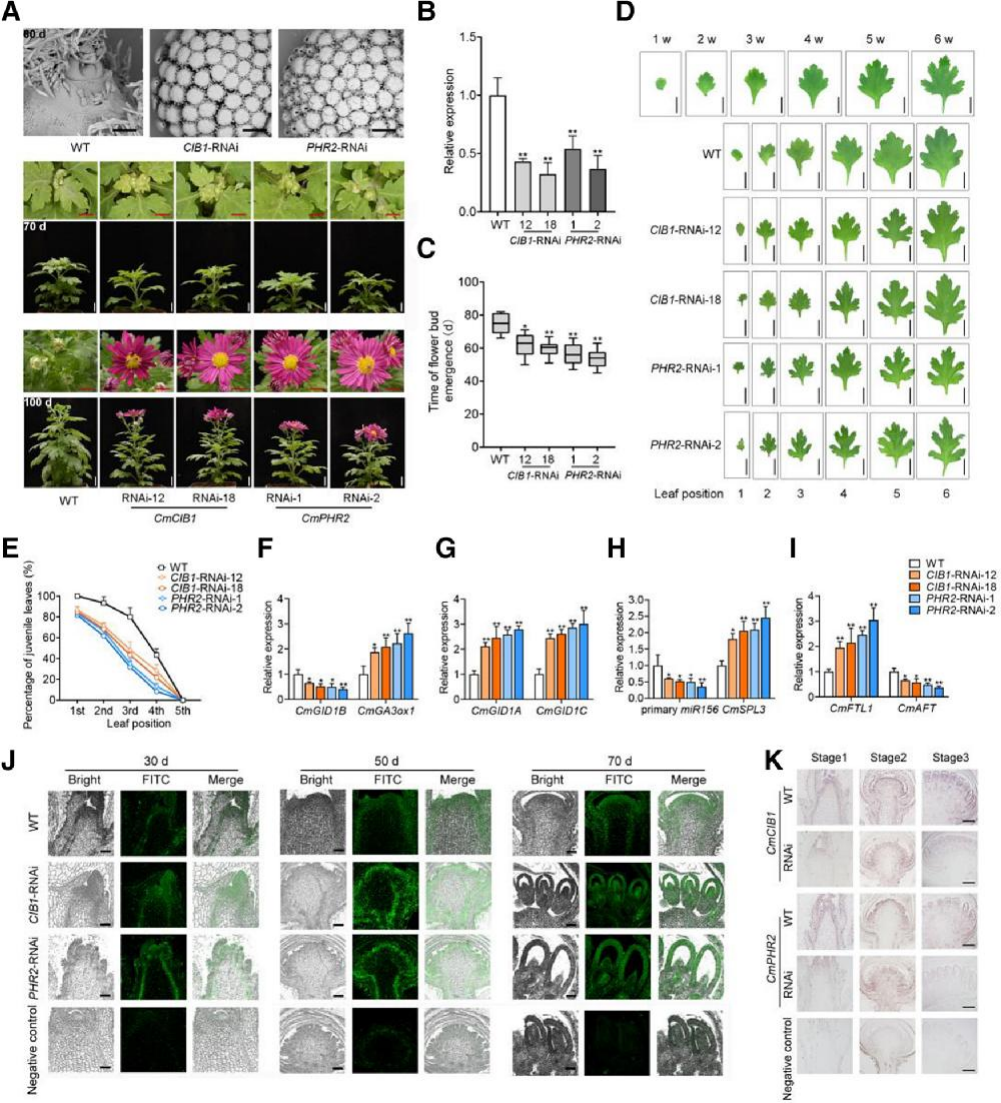
*CmCIB1-*RNAi and *CmPHR2-*RNAi plants have a shorter juvenile vegetative phase and flower early. **A)** Shoot apices observed by scanning electron microscopy (sem) (top row) and phenotypes (lower rows) of WT, *CmCIB1-*RNAi, and *CmPHR2-*RNAi plants at 60 d under LD conditions. Scale bars (sem images), 100 *μ*m. Scale bars (shoot apex images), 1 cm. Scale bars (front view images of plant), 2 cm. **B)** Relative expression levels of *CmPHR2* and *CmCIB1* analyzed by RT-qPCR in WT, *CmCIB1-*RNAi, and *CmPHR2-*RNAi plants grown for 40 d under LD conditions. **C)** Time of flower bud emergence of WT, *CmCIB1-*RNAi, and *CmPHR2-*RNAi plants. Eighteen samples were used to calculate the days of flower bud emergence; *n* = 18. Center line, median; box limits, upper and lower quartiles; whiskers, 1.5× interquartile range; points, outliers. **D)** Morphology of leaves of WT, *CmCIB1-*RNAi, and *CmPHR2-*RNAi chrysanthemum plants grown under LD conditions for 30 d. Samples were photographed at the same time, and images were digitally extracted for comparison. Scale bars, 1 cm. **E)** Percentage of juvenile leaves among the first 5 leaves in WT, *CmCIB1-*RNAi, and *CmPHR2-*RNAi chrysanthemum plants. **F** to **I)**. Relative expression levels of *CmGID1B* and *CmGA3ox1***F)**, *CmGID1A* and *CmGID1C***G)**, primary *cmo-miR156* and *CmSPL3***H)**, and *CmFTL1* and *CmAFT***I)** analyzed by RT-qPCR in WT, *CmCIB1-*RNAi, and *CmPHR2-*RNAi plants grown for 40 d. The results are the means of 3 biological replicates with standard deviation in **B**, **E**, **F** to **I)**. Asterisks indicate significant differences according to a Student's *t*-test in **B**, **C**, **E**, **F** to **I)** (*P < 0.05, **P < 0.01). **J)** Immunolocalization of GA in the shoot apices of WT, *CmCIB1-*RNAi, and *CmPHR2-*RNAi plants at different stages of floral transformation, with a polyclonal anti-GA antibody. Green fluorescence is the immune signal. For the negative controls, the antibody was omitted. Scale bars, 200 *μ*m. **K)** In situ hybridization analysis of *CmPHR2* and *CmCIB1* transcripts (shown in purple) in the apical meristems of WT, *CmCIB1-*RNAi, and *CmPHR2-*RNAi plants at different stages of floral transformation. The negative control was hybridized with the sense probe. Scale bars, 200 *μ*m.

We investigated whether CmCIB1 and CmPHR2 regulate aging and flowering by generating their knockdown lines, *CmCIB1*-RNAi and *CmPHR2*-RNAi (Fig. 6B). The phenotypes of *CmCIB1*-RNAi and *CmPHR2*-RNAi plants were similar to those of *CmGID1B*-RNAi plants. Indeed, both *CmCIB1*-RNAi and *CmPHR2*-RNAi plants were able to flower without SD induction. Microscopy observations also showed that their apical meristems enter the early stage of floret primordium differentiation after 55 d in LD conditions, while WT plants remained at the vegetative stage (Fig. 6, A and C).

We observed flower bud emergence in *CmCIB1*-RNAi plants and *CmPHR2*-RNAi plants within 60 d, but not in WT plants grown under the same conditions (Fig. 6A). Blooming of flower buds occurred within 95 d in *CmCIB1*-RNAi and *CmPHR2*-RNAi plants, while the WT plants were still at the flower bud development stage (Fig. 6A).

We also inspected leaf morphology and scored the proportion of juvenile leaves from WT, *CmCIB1*-RNAi, and *CmPHR2*-RNAi plants. All first-emerging leaves, 96% of second leaves, and 82% of third leaves from WT plants were typically juvenile. However, more than 16%, 31%, and 55% of first-, second-, and third-emerging leaves, respectively, showed an adult morphology in *CmCIB1*-RNAi lines. Likewise, more than 18%, 34%, and 65% of first-, second-, and third-emerging leaves, respectively, showed an adult morphology in *CmPHR2*-RNAi lines (Fig. 6, D and E). These results indicate that CmCIB1 or CmPHR2 regulates the transition time from the juvenile phase to the adult phase in chrysanthemum.

To connect CmCIB1 and CmPHR2 with *CmGID1B* expression in the modulation of flowering by aging, we evaluated the expression of *CmGID1B* in the *CmCIB1*-RNAi and *CmPHR2*-RNAi lines. Importantly, *CmGID1B* was significantly downregulated in both sets of RNAi lines (Fig. 6F). The expression of primary *cmo*-*miR156* and *CmAFT* was also significantly downregulated, while that of *CmSPL3* and *CmFTL1* was significantly upregulated in both *CmCIB1*-RNAi and *CmPHR2*-RNAi lines relative to the WT. These expression patterns were generally consistent with those seen in the *CmGID1B*-RNAi lines (Fig. 6, H and I).

We also evaluated the expression of *CmGA3ox1*, *CmGID1A,* and *CmGID1C,* which were significantly upregulated in both *CmCIB1*-RNAi and *CmPHR2*-RNAi lines (Fig. 6, F and G). In addition, immunolocalization of GAs detected a stronger signal in both *CmCIB1*-RNAi and *CmPHR2*-RNAi plants than in the WT. We established that the accumulation of *CmCIB1* and *CmPHR2* transcripts is consistent with that of *CmGID1B* mRNA and GAs based on in situ hybridization (Figs. 4, D and E and 6, J and K).

In addition, we transiently overexpressed *CmCIB1* and *CmPHR2* in chrysanthemum, respectively, and found that both overexpression lines showed a substantially early flowering phenotype. The expression of primary *cmo*-*miR156* and *CmAFT* was substantially downregulated, while that of *CmSPL3* and *CmFTL1* was substantially upregulated in both *pSuper:*CmCIB1-GFP and *pSuper:*CmPHR2-GFP plants relative to the control plants (Supplemental Fig. S3).

Together, these results demonstrate that the CmCIB1-PHR2 complex activates the expression of *CmGID1B* in response to SDs, thereby regulating the biosynthesis and signal transduction of endogenous GAs and thus enabling the transition from the juvenile stage to the adult and flowering stages by affecting the genes of the aging pathway in chrysanthemum.

## Discussion

**Figure 7.**
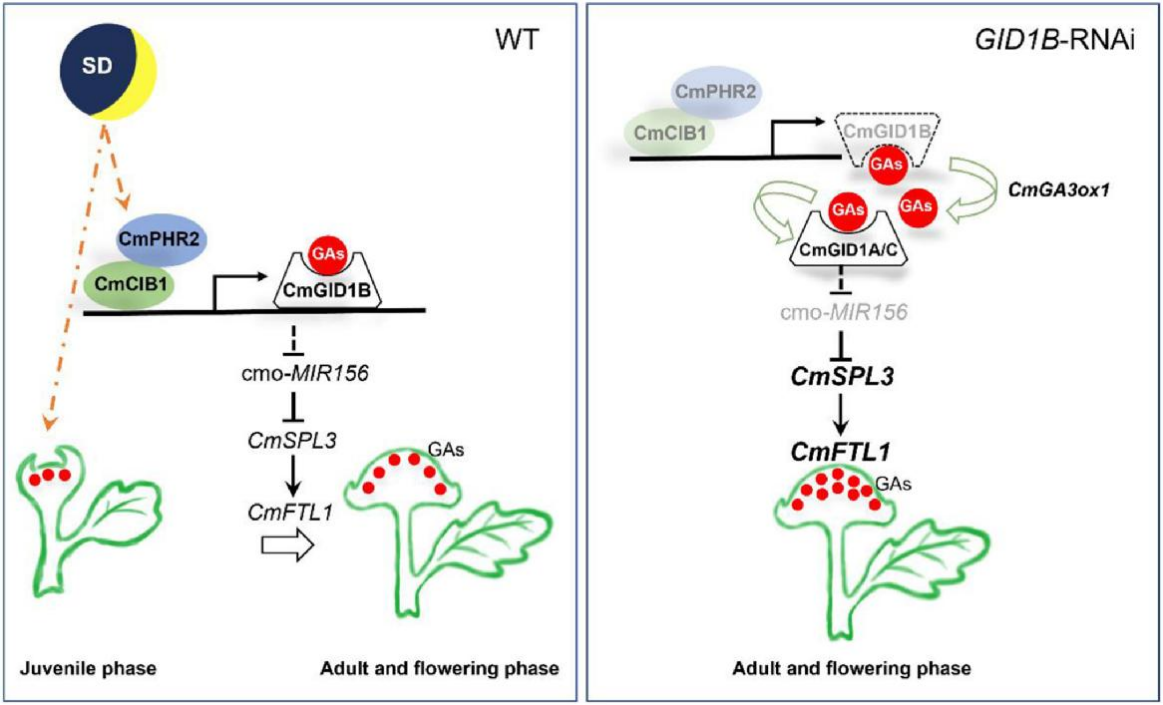
Schematic model of CmGID1B-mediated integration of photoperiodic signals and the aging pathway regulating the floral transition in chrysanthemum. In juvenile WT plants, the increase of endogenous GA contents and raised CmPHR2-CIB1 complex promoted the expression of *CmGID1B* and activated GA signal in response to SD. Therefore, *miR156* expression was inhibited, and *CmSPL3* transcription level was upregulated to initiate the transition from seedlings to adults and finally promoted the flowering transition through activation of *CmFTL1* transcription. In *CmGID1B*-RNAi plants, there are more bioactive GA contents and higher expression of *CmGID1A* and *CmGID1C*, and the enhanced perception of GA signals further inhibits *miR156* expression, resulting in increased transcription levels of *CmSPL3* and *CmFTL1*, which accelerated the transition from juvenile to adult and flowering. The number and location of red dots represent the contents and distribution of active GAs, respectively. The dotted line represents indirect regulation, and the solid line represents direct regulation. The lighter color of the same protein or the smaller font size of the same gene represents the lower expression level, and the darker color or the thicker font size represents the higher expression level.

Plants acquire the ability to flower and reach reproductive development during the juvenile-to-adult transition, and plants in the adult phase respond to both environmental cues and endogenous signals to complete the floral transformation and achieve reproductive success (Huijser and Schmid 2011; Srikanth and Schmid 2011). Photoperiod is a critical environmental cue for many plants, such as Arabidopsis (Cheng and Wang 2005), rice (Kim et al. 2008), soybean (*Glycine max*) (Luo et al. 2021), and chrysanthemum (Higuchi et al. 2013). Genetic analyses have identified the plant hormone GA as having a prominent role in the regulation of flowering time (Sun 2011). We previously reported that CmBBX24 acts as a core repressor that prevents chrysanthemum flowering by inhibiting GA biosynthesis under LD conditions (Yang et al. 2014), but little is known about whether SD also affects GA signaling and/or biosynthesis. The aging pathway is generally thought to determine flowering via an internal developmental program in plants, with few reports indicating whether it is also influenced by environmental cues. A recent study found that FAR-RED ELONGATED HYPOCOTYL3 and FAR-RED IMPAIRED RESPONSE1 directly interact with SPL3, SPL4, and SPL5 and downregulate *LFY* and *AP1* expression, thus delaying flowering in response to shade conditions (Xie et al. 2020). However, it has not been experimentally tested whether and how the aging pathway mediated by the miR156-SPL module regulate flowering in response to changes in photoperiod and/or GA signal perception. Our current results provide a molecular framework: when juvenile WT plants were exposed to SD conditions, the increased endogenous GA contents and raised CmPHR2-CIB1 complex promoted *CmGID1B* expression, thus activating GA signaling to downregulate *miR156* expression and upregulating *CmSPL3* transcript levels to initiate the juvenile-to-adult transition and finally promote floral transition by activating *CmFTL1* transcription. However, there are more bioactive GA contents and higher expression of *CmGID1A* and *CmGID1C* in *CmGID1B*-RNAi plants, and the enhanced perception of GA signals further inhibits *miR156* expression, resulting in higher *CmSPL3* and *CmFTL1* transcript levels, and accelerates the juvenile-to-adult transition and flowering (Fig. 7).

The photoperiodic pathway begins when photoreceptors sense photoperiod and transmit signals to downstream regulatory networks (Cashmore 2003). Of the 3 types of photoreceptors identified in plants, CRYs mainly absorb blue and ultraviolet light, and Arabidopsis CRY1 and CRY2 interact with CIB1 and CO to promote flowering (Liu et al. 2010, 2018). Here, we showed that the atypical photolyase/blue light photoreceptor CmPHR2, but not CRYs, responds to SDs and interacts with CmCIB1 to regulate flowering in chrysanthemum. PHR2 was previously identified in Arabidopsis (Ahmad and Jarillo 1998), but a role in flowering remained unclear. In this study, we detected the upregulation of *CmCIB1* and *CmPHR2* expression when chrysanthemum was exposed to SD conditions. We established that CmCIB1 activates *CmGID1B* transcription by binding directly to the *CmGID1B* promoter (Fig. 5). Furthermore, the formation of the CmCIB1-PHR2 complex enhances the activation of *CmGID1B* promoters, and we speculate that CmPHR2 may promote the stability of CmCIB1 protein. According to previous reports, blue light receptors ZEITLUPE and LOV KELCH PROTEIN 2 inhibit CIB1 ubiquitination degradation under blue light (Liu et al. 2013). Knocking down *CmCIB1* or *CmPHR2* resulted in a similar earlier flowering in chrysanthemum (Fig. 6, A to C). Hence, we conclude that CmCIB1-PHR2 is an upstream regulator of *CmGID1B* transcription that plays a key role in regulating flowering time in response to SD conditions.

There is a direct association between the GA signaling and endogenous GA level in plants (Ge and Steber 2018). Indeed, the accumulation of GAs activates the GA signal by promoting the GID1-dependent degradation of DELLA, but the excessive accumulation of GAs can also result in lower GID1 abundance (Ge and Steber 2018), which contributes to an important feedback system controlling GA signals. A previous study suggested that GID1A, GID1B, and GID1C are partially redundant in their regulation of flowering in Arabidopsis (Nakajima et al. 2006), as evidenced by the gradual delay from single mutants to the triple mutant, which completely lost the ability to flower (Griffiths et al. 2006). Arabidopsis GID1s appear to exhibit some specificity in their regulation of filament and silique elongation (Jiang et al. 2017; Hauvermale and Steber 2020), with GID1A involved in silique elongation, while GID1C participates in filament elongation (Griffiths et al. 2006). In addition, how GID1 receptors regulate growth and development may also be specialized, depending on their binding affinity with DELLA proteins (Griffiths et al. 2006; Yamaguchi et al. 2014). Recent studies have shown that CRY1 interacts with GID1s in a blue light–dependent manner, and the CRY1–GID1 interaction promotes DELLA accumulation (Liu et al. 2010; Zhong et al. 2021). In our work, *CmGID1B* transcription was directly activated by the CmCIB1-PHR2 module in response to SDs, concomitantly with an increase in endogenous GA contents, resulting in an activation of the GA cascade to promote floral transition in chrysanthemums. Interestingly, we discovered a characteristic of GID1 receptors that manifested in *CmGID1B*-RNAi plants, which accumulated more GA_1_ and GA_4_, while also exhibiting stronger GA signal perception due to higher transcriptional abundance of *CmGID1A* and *CmGID1C*, leading to the shortening of the juvenile stage of these transgenic plants. However, the flowering time of plants that silenced all 3 *CmGID1s* was substantially delayed. We consider that CmGID1A/B/C had complementary functions for GA perception, and CmGID1B is more critical to the regulation of the floral transition in chrysanthemum.

It has been reported that miR156 plays a critical role in the aging pathway by directly targeting transcripts from the SPL family of transcriptional regulators (Wu and Poethig 2006; Wang et al. 2009). DELLAs directly inhibit the transcriptional activation of *SPL*s and interact with SPL to inhibit their transcriptional activity to negatively regulate downstream flowering genes such as *SOC1* and *FUL* (Yu et al. 2012). However, in the course of flower formation, DELLAs also activate *AP1* transcription by interacting with SPL9 to promote the initial formation of the floral primordium (Hyun et al. 2016). Therefore, DELLAs may recruit different SPLs to target various downstream targets, thus allowing GAs to act positively and then negatively to control the onset of floral transformation in Arabidopsis. Here, we found that *cmo-miR156* transcript levels are also regulated by GA signaling in response to SD and then target *CmSPL3* to regulate the floral transition in chrysanthemum. The expression of primary *cmo-miR156* was substantially downregulated in *CmCIB1*-RNAi, *CmPHR2*-RNAi, and *CmGID1B*-RNAi plants and was accompanied by the upregulation of *CmSPL3* transcript levels. The transgenic plants therefore completed the juvenile-to-adult transition faster and initiated floral transition before WT plants.

## Materials and methods

### Plant materials and growth conditions

*CmGID1A*-RNAi, *CmGID1B*-RNAi, *CmGID1C*-RNAi, *CmCIB1*-RNAi, *CmPHR2*-RNAi, and WT chrysanthemum (*C. morifolium* “Fall Color”) were used in this study. To construct the RNAi vector, a 459-bp sense and antisense fragment of *CmGID1A*, a 483-bp sense and antisense fragment of *CmGID1B*, a 467-bp sense and antisense fragment of *CmGID1C*, a 549-bp sense and antisense fragment of *CmCIB1*, and a 472-bp sense and antisense fragment of *CmPHR2* were respectively cloned into the pFGC1008 vector by AscI/SwaI or BamHI/PacI sites to obtain an intron-containing “hairpin” RNA construct with 35S promoter. The recombinant vectors were transferred into *Agrobacterium tumefaciens* strain EHA105, and the chrysanthemum leaf disc was used as explant for infection. The resistant plants were screened by hygromycin. The primers used are shown in Supplemental Table S1.

Forty-day-old plants were transplanted into 9 cm diameter pots filled with a peat: vermiculite (1:1, v/v) mixture and grown in a culture room at 23 ± 1 °C, with 40% relative humidity, 100 *μ*molm^−2^ s^−1^ illumination, a LD photoperiod of 16 h light/8 h dark, and a SD photoperiod of 8 h light/16 h dark.

### RNA extraction and RT-qPCR

The upper 4th expanded leaf was collected from 5 biological replicates at Zeitgeber time 8. Total RNA was extracted from the samples mentioned above using the RNAiso Plus reagent (TaKaRa, Japan) according to the manufacturer's instructions. cDNAs were synthesized from 1 *μ*g total RNA using the HiScript II Q RT SuperMix for qPCR (+gDNA wiper) (Vazyme, Nanjing, China). The RT-qPCR was carried out using the StepOne Real-Time PCR System (Applied Biosystems, USA) in its standard mode and the 2× Realtime PCR Super Mix (SYBR green, with anti-Taq) (Mei5 Biotechnology Co., Ltd., China). The chrysanthemum *UBIQUITIN* gene (GenBank accession: NM_112764) was used as an internal control. Relative expression levels were calculated using the 2^−ΔΔCT^ method (Livak and Schmittgen 2001). The gene-specific primers are listed in Supplemental Table S1.

### Determination of GA contents

Terminal shoots were taken from WT and *CmGID1B*-RNAi tissue culture seedlings grown under LDs for 40 d in 3 replicates. GA contents were determined by a commercial company (Metware Biotechnology Co., Ltd., Wuhan, China) using the AB Sciex QTRAP 6500 liquid chromatography-tandem MS (LC-MS/MS) platform. Raw data were analyzed by software Analyst 1.6.3 (AB Sciex, Waltham, MA, USA).

### GA treatment

WT and *CmGID1B*-RNAi tissue culture seedlings grown in LD for 40 d were transferred to the LD culture chamber for 2 wk and then transferred to SD culture and sprayed with 100 *μ*m GAs. GA_1_ or GA_4_ was dissolved in 3% (v/v) DMSO, and the same concentration of DMSO was used as the control. Plants were sprayed every 5 d for 1 mo.

### Subcellular localization

The *CmGID1A*, *CmGID1B*, and *CmGID1C* ORF sequences (without terminator) were fused with GFP to construct the pCAMBIA-1300 vector by PacI/BamHI sites, respectively. The fusion protein was expressed in chrysanthemum mesophyll cell protoplasts for subcellular localization analysis of CmGID1B, and empty GFP was used as a control. After the transformed protoplasts were cultured in the dark for 18 h, the fluorescence signal was detected by a Nikon A1 confocal laser scanning microscope (Nikon, Japan). GFP was excited using a 488-nm laser line with the detection wavelength from 525 nm. RFP was excited using a 561-nm laser line with the detection wavelength from 610 nm. The primers used are shown in Supplemental Table S1.

### Phenotypic measurements

To evaluate the time until initial flower bud emergence, the day of transplanting was set as day 1. The time of the 1st visible flower buds (2 mm diameter) was then recorded. The shoot apex and inflorescence were dissected from the chrysanthemum under a light microscope (Leica DFC450, Germany). After dissection, samples were immediately observed by scanning electron microscopy (Hitachi TM4000, Japan) with an accelerating voltage of 15 kV.

### Virus-induced gene silencing

To silence *CmGID1s* in chrysanthemum, a previously reported virus-based microRNA expression system was used (Tang et al. 2010; Xu et al. 2020). A modified CaLCuV vector containing pre-cmo-CmGID1s (CaLCuV + CmGID1s) was generated and introduced into the *A. tumefaciens* strain GV3101. The transformed *A. tumefaciens* cultures were inoculated overnight in LB medium and resuspended in infiltration buffer to a final OD_600_ of 2. Then, the cultures containing pCVB and CaLCuV-GID1, or pCVB and CaLCuV (control), were mixed in a 1:1 ratio (v/v) and incubated in the dark at 28 °C for 3 to 4 h before vacuum infiltration. Fifty-day-old WT plants were immersed in infiltration buffer using a needleless syringe. Then, the plants were placed in the dark at 8 °C for 3 d and transplanted into pots filled with a mixture of peat: vermiculite (1:1, v/v) and grown at 23 ± 1 °C under LD conditions. The silenced plants were validated by RT-qPCR to detect the expression of *CmGID1*. Three independent experiments were performed, and at least 6 positive plantlets were used to observe the phenotypes.

### RNA-seq analysis

WT and *CmGID1B*-RNAi tissue culture seedlings grown in LD for 40 d were transferred to the LD culture chamber for 2 wk and then transferred to SD culture chamber for 2 wk. When the growth point was almost transformed into a floral primordium, the top 4th expanded leaf was taken from 3 biological replicates to extract total RNA. RNA-Seq libraries (Zhong et al. 2011) were prepared and sequenced using HiSeq 2000 (Illumina) platform at the Novogene Co. Ltd (Beijing, China, http://www.novogene.com/). RNA-seq data were processed, assembled, and annotated as previously described (Wei et al. 2017).

### In situ hybridization

Shoot apices were fixed in 3.7% (v/v) formalin acetic alcohol overnight and then dehydrated using a series of gradient ethanol solutions (30%, 50%, 70%, 80%, 90%, and 100% [v/v]). After dehydration, samples were transferred to xylene and then paraplast (Leica, Germany) and coated in 100% (v/v) paraplast. A microtome (HistoCore BIOCUT, Leica Biosystems) was used to cut the embedded samples into 10 *μ*m sections. *CmGID1B* probes were designed according to the 3′ UTR specific region. Sense and antisense probes were synthesized using SP6 and T7 RNA polymerase, respectively. In situ hybridization experiments were performed as previously described (Zhang et al. 2013). The primers used are shown in Supplemental Table S1.

### Immunolocalization of GA

Shoot apices were fixed in 3.7% (v/v) formalin acetic acid and prepared into paraffin sections. Paraffin sections were incubated with a 1:50 (v/v) dilution of polyclonal antibody to Gibberellic Acid (Immuno Clone Biosciences Co., Ltd, USA) overnight at 4 °C and then with a 1:200 (v/v) dilution of Goat AntiRabbit IgG HandL (Alexa Fluor 488) (Abcam, China) for 4 h at room temperature in the dark. Fluorescence signals were recorded using a Nikon A1 confocal laser scanning microscope (Nikon, Japan) with an excitation wavelength of 488 nm and an emission wavelength of 519 nm. Negative controls were specimens not incubated with anti-GA antibodies.

### Y1H assay

The Matchmaker Gold Yeast single Hybrid Library Screening System (Clontech, Japan) was used to determine protein and DNA interactions in yeast (*Saccharomyces cerevisiae*) cells. To identify the upstream genes of *CmGID1B*, we constructed a 1-hybrid library using high-quality chrysanthemum cDNA. The promoter sequence of *CmGID1B* was then divided into 3 fragments and inserted into the pAbAi vector (Clontech, Japan). According to the results of yeast single hybrid screening library, we selected CmCIB1. To test whether CmCIB1 binds to the promoter of *CmGID1B*, the full-length sequences of *CmCIB1* were inserted into the pGADT7 vector by EcoRI/BamHI sites, and the *CmGID1B* promoter fragments were inserted into the pAbAi vector by KpnI/XhoI sites. Interaction assays were performed with 200 mg/*µ*L aureobasidin A (AbA) (Clontech, Japan) on strictly selected SD/-Ura/-Leu medium. The rimers used are shown in Supplemental Table S1.

### Dual-luciferase reporter assay in *N. benthamiana*

To investigate whether CmCIB1 and CmPHR2 directly regulate *CmGID1B* in vivo, we used pGreenII 0800-LUC and pGreenII 0029 62-SK vectors (Hellens et al. 2000). The 1341 bp *CmGID1B* promoter sequence was inserted into pGreenII 0800-Luc vector by the HindI/BamHI sites. The coding sequences of *CmCIB1* and *CmPHR2* were inserted into pGreenII 62-SK vector by EcoRI/KpnI sites. The recombinant vectors were introduced into *A. tumefaciens* strain GV3101 containing the pMP90 and pSoup plasmids (Hellens et al. 2005). Mixtures of *A. tumefaciens* cultures expressing coding sequence or the promoter fragments (v:v, 1:5) were infiltrated into *N. benthamiana* leaves using a needleless syringe (Wei et al. 2017). LUC and REN activities were measured using dual-luciferase reporter assay reagents (Promega, USA) and a GloMax 20/20 luminometer (Promega, USA). The ratios of LUC and REN were expressed as activation or repression. The LUC images were taken using an iKon-L936 imaging system (Andor Tech, Belfast, UK). The primers used are shown in Supplemental Table S1.

### Transient overexpression

The full-length sequences of *CmGID1B, CmCIB1*, and *CmPHR2* without stop codon were inserted into pSuper1300 (GFP-C) vector by XbaI/KpnI sites. The obtained construct and empty vector control were separately introduced into *A. tumefaciens* strain GV3101. Subsequently, *Agrobacterium* cultures were collected by centrifugation and resuspended in infiltrating buffer (10 mm MES, 10 mm MgCl_2_, 200 mm AS, and pH 5.8) to a final OD_600_ of 1.0 and infiltrated into chrysanthemum leaves using a needleless syringe. Then, the plants were placed in the dark at 8 °C for 3 d and transplanted into pots filled with a mixture of peat: vermiculite (1:1, v/v) and grown at 23 ± 1 °C under LD conditions. The overexpressed plants were validated by RT-qPCR to detect the expression of *CmGID1B, CmCIB1*, and *CmPHR2*. Three independent experiments were performed, and at least 6 positive plantlets were used to observe the phenotypes. The primers used are shown in Supplemental Table S1.

### ChIP assay

ChIP experiments were performed according to the general protocol described previously (Saleh et al. 2008). Approximately 3 g of young leaves were frozen and ground in liquid nitrogen, crosslinked with 1% (v/v) formaldehyde for 10 min, terminated by adding 0.125 mm glycine for 5 min, extracted with chromatin and sonicated, and followed by immunoprecipitation using the anti-GFP antibody (BE2001, Easybio, Beijing, China) and Magna ChIP Protein A + G Magnetic Beads (EMD Millipore, USA) overnight. The coprecipitated DNA was purified with a QIAquick PCR Purification Kit (Qiagen GmbH, Germany). The enrichment degree of DNA fragments was detected by RT-qPCR. The primers used are shown in Supplemental Table S1.

### Y2H assays

The Y2H assay was conducted using the Matchmaker GAL4 2-hybrid system (Clontech, Shiga-ken, Japan). The *CmCIB1* ORF sequence was amplified and inserted into the pGADT7 vector by EcoRI/BamHI sites (Chien et al. 1991). The *CmPHR2* ORF sequence was amplified and inserted into the pGBKT7 vector by EcoRI/SalI sites (Louvet et al. 1997). The pGADT7 and pGBKT7 recombinant plasmids were cotransformed into yeast strain Y2HGold. The *GUS* ORF sequence was inserted into the pGADT7 vector or the pGBKT7 vector as a negative control. Transformants were grown on SD/-Trp-Leu plates and then transferred to SD/-Trp-Leu-His plates with X-α-gal for spot analysis. The primers used are shown in Supplemental Table S1.

### BiFC

The *CmCIB1* ORF without terminator was inserted into the 35S-SPYCE(M) vector by XbaI/KpnI sites, the *CmPHR2* ORF was inserted into the 35S-SPYNE(R)173 vector by XbaI/KpnI sites, and the recombinant vectors or control vectors were transferred into *A. tumefaciens* strain GV3101. The OD600 was adjusted to 1.0 with infiltration buffer. Mixtures of *A. tumefaciens* cultures expressing CmPHR2-YFP^N^, CmCIB1-YFP^C^, or control vectors were mixed in a 1:1 ratio (v/v) and were infiltrated into *N. benthamiana* leaves using a needleless syringe After 3 d, YFP fluorescence was imaged using a Nikon A1 confocal laser scanning microscope (Nikon, Japan). YFP was excited using a 488-nm laser line with the detection wavelength from 525 nm. RFP was excited using a 561-nm laser line with the detection wavelength from 610 nm. The primers used are shown in Supplemental Table S1.

### Pull-down assays

The *CmCIB1* ORF sequence was amplified and inserted into the pET-28a vector by BamHⅠ/EcoRⅠ sites. The *CmPHR2* ORF sequence was amplified and inserted into the pGEX-4T-2 vector by BamHI/EcoRI sites. The recombinant vectors were transferred into the expression competent *Efficom* GSsetta (DE3) (Beijing Genesand Biotech Co., Ltd, China), and the proteins were induced by 0.2 mm IPTG at 16 °C overnight. The GST-CmPHR2 protein and GST protein were purified as the bait proteins using the Glutathione Sepharose 4 Fast Flow (GE Healthcare Life Sciences, USA), and the His-CmCIB1 protein was purified using the Ni Sepharose 6 Fast Flow (GE Healthcare Life Sciences, USA). Then, the His-CmCIB1 protein as the prey protein was incubated with the GST-CmPHR2 protein and GST protein overnight at 4 °C. Bait proteins and prey proteins were detected by anti-GST (Beyotime Biotechnology, China) and anti-His (CoWin Biosciences, China), respectively. Protein bands were detected by Bioanalytical Imaging System (Azure Biosystems, Inc, USA). The primers used are shown in Supplemental Table S1.

### Statistical analysis

At least 3 biological replicates were included in the data, and the statistical significance of differences was determined by ANOVA followed by the Duncan's multiple range test or the Student's *t*-test (GraphPad Prism version 8).

### Accession numbers

Sequence data from this article can be found in the National Center for Biotechnology Information databases under the following accession numbers: *CmGID1B* (OP889328), *CmCIB1* (OP889329), *CmPHR2* (OP889330), *CmSPL3* (KT253091), *CmFTL1* (AB545936), and *CmGA20ox* (ABQ17965).

## Supplementary Material

kiad503_Supplementary_Data

## Data Availability

All data included in this study are available upon request by contact with the corresponding author.

## Supplemental data

The following materials are available in the online version of this article.

**Supplemental Figure S1.** GA regulates the floral transition of chrysanthemums.

**Supplemental Figure S2.** Subcellular localization analysis of CmGID1s and diurnal rhythm analysis of *CmGID1s* expression.

**Supplemental Figure S3.** Transient overexpression of *CmCIB1*, *CmPHR2*, and *CmGID1B* plants flowering early.

**Supplemental Figure S4.** Relative expression of the other 2 *GID1* members in *CmGID1A*-RNAi or *CmGID1C*-RNAi plants.

**Supplemental Figure S5.** Relative expression levels of primary *cmo-miR156*, *CmSPL3*, and *CmAFT* with GA treatment.

**Supplemental Figure S6.** Diurnal rhythm analysis of *CmCIB1* and *CmPHR2* expression and subcellular localization analysis of CmCIB1 and CmPHR2.

**Supplemental Table S1.** Primers used in this study.

**Supplemental Data Set 1.** Differentially transcribed genes (DTGs) in *CmGID1B*-RNAi and control plants.

## Dive Curated Terms

The following phenotypic, genotypic, and functional terms are of significance to the work described in this paper:

CIB1 Gramene: AT4G34530
CIB1 Araport: AT4G34530

CRY2 Gramene: AT1G04400
CRY2 Araport: AT1G04400

lkp2 Gramene: AT2G18915
lkp2 Araport: AT2G18915

SPL3 Gramene: AT2G33810
SPL3 Araport: AT2G33810

SPL4 Gramene: AT1G53160
SPL4 Araport: AT1G53160

SPL5 Gramene: AT3G15270
SPL5 Araport: AT3G15270

SPL9 Gramene: At2g42200
SPL9 Araport: At2g42200

CIB5 Gramene: AT1G26260
CIB5 Araport: AT1G26260

CRY3 Gramene: AT5G24850
CRY3 Araport: AT5G24850

CRY1 Gramene: AT4G08920
CRY1 Araport: AT4G08920

ZTL Gramene: AT5G57360
ZTL Araport: AT5G57360

GID1a Gramene: AT3G05120
GID1a Araport: AT3G05120

GID1b Gramene: AT3G63010
GID1b Araport: AT3G63010

GID1c Gramene: AT5G27320
GID1c Araport: AT5G27320

BBX24 Gramene: At1g06040
BBX24 Araport: At1g06040
