# Supplementary material for: PHOTOLYASE/BLUE LIGHT RECEPTOR2 regulates chrysanthemum flowering by compensating for gibberellin perception
Source: Plant Physiol. 2023 Sep 19;193(4):2848–64. doi: 10.1093/plphys/kiad503 (PMC10663108; doi:10.1093/plphys/kiad503)
Supplement: kiad503_Supplementary_Data [file kiad503_supplementary_data.zip › Supplemental Data.pdf]

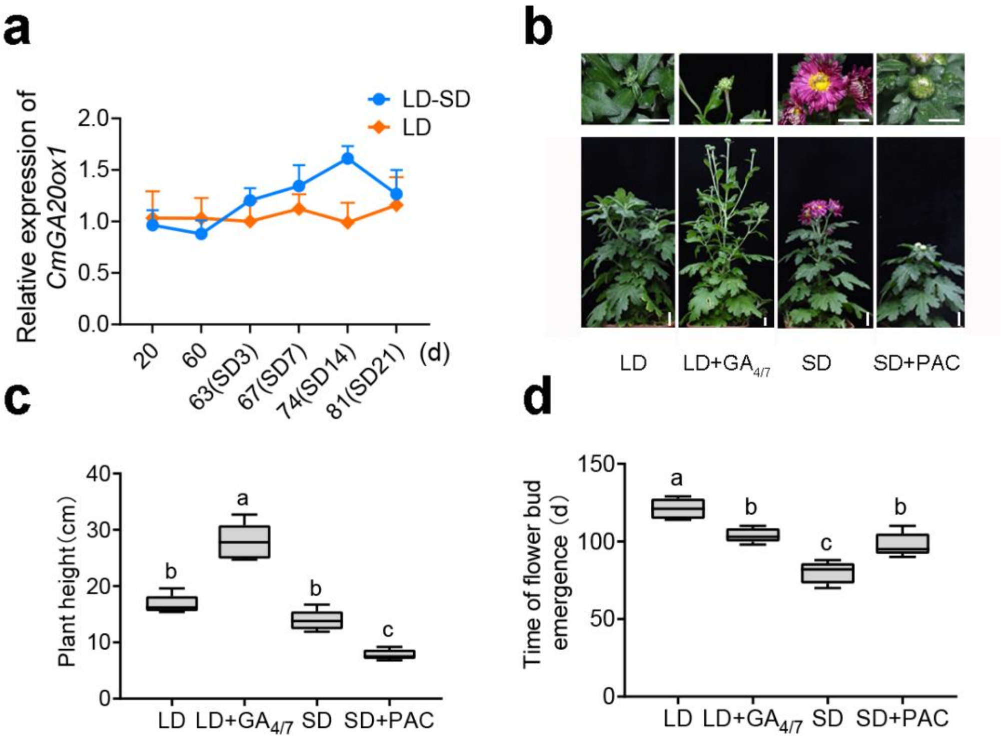

Supplemental Figure S1. GA regulates the floral transition of chrysanthemums. a. Expression of *CmGA20ox1* was analyzed by RT-qPCR in chrysanthemum from vegetative growth to flowering transition. *UBIQUITIN* was used as an internal control. The results are the means of three biological replicates with standard deviation. b. Phenotypes of WT plants treated with 100 μM $GA_{4/7}$ under LD conditions or treated with 100 μM PAC under SD conditions. Scale bars, 1 cm. c-d. Plant height (c) and time of flower bud emergency (d) of WT plants treated with 100 μM $GA_{4/7}$ under LD conditions or treated with 100 μM PAC under SD conditions. 12 samples were used to calculate the plant height and days of flower bud emergence; n=12. Center line, median; box limits, upper and lower quartiles; whiskers, 1.5× interquartile range; points, outliers. Different lowercase letters indicate significant differences according to Duncan's multiple range test in c, d ($P < 0.05$).

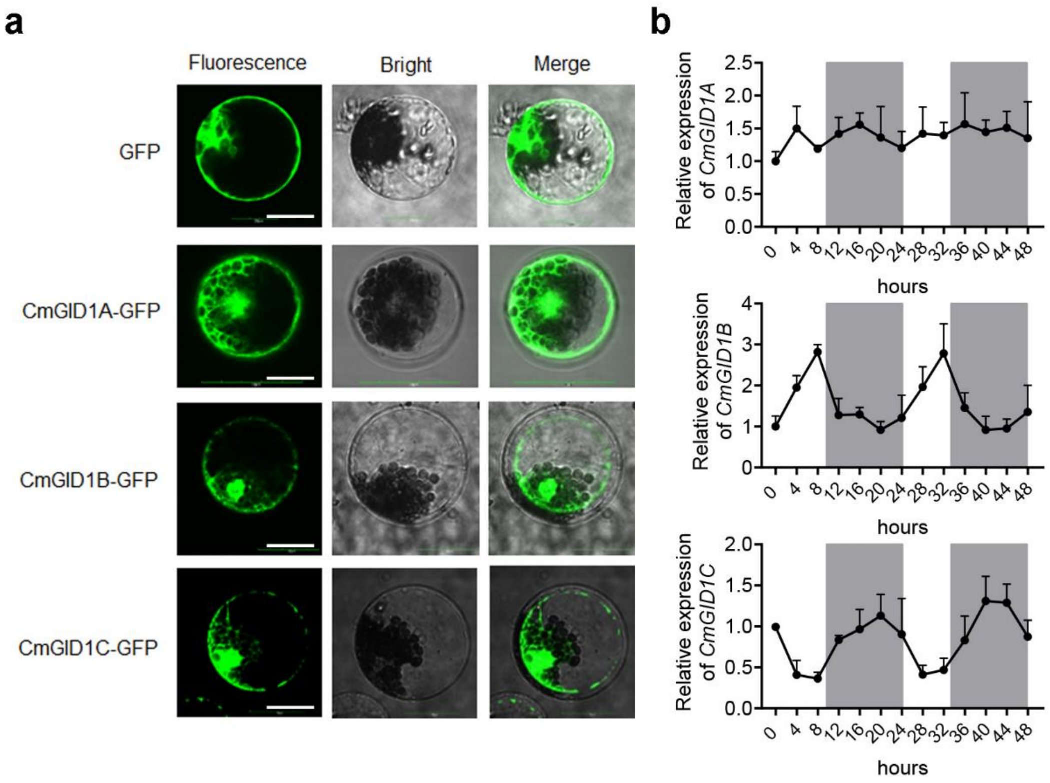

Supplemental Figure S2. Subcellular localization analysis of CmGID1s and diurnal rhythm analysis of *CmGID1s* expression. a. Subcellular localization analysis of CmGID1s. The empty GFP was used as a control. The green fluorescence is the GFP signal. Scale bars, 20 µm. b. Diurnal rhythm expression of *CmGID1A-C* within 48 hours under SD conditions. The results are the means of three biological replicates with standard deviation.

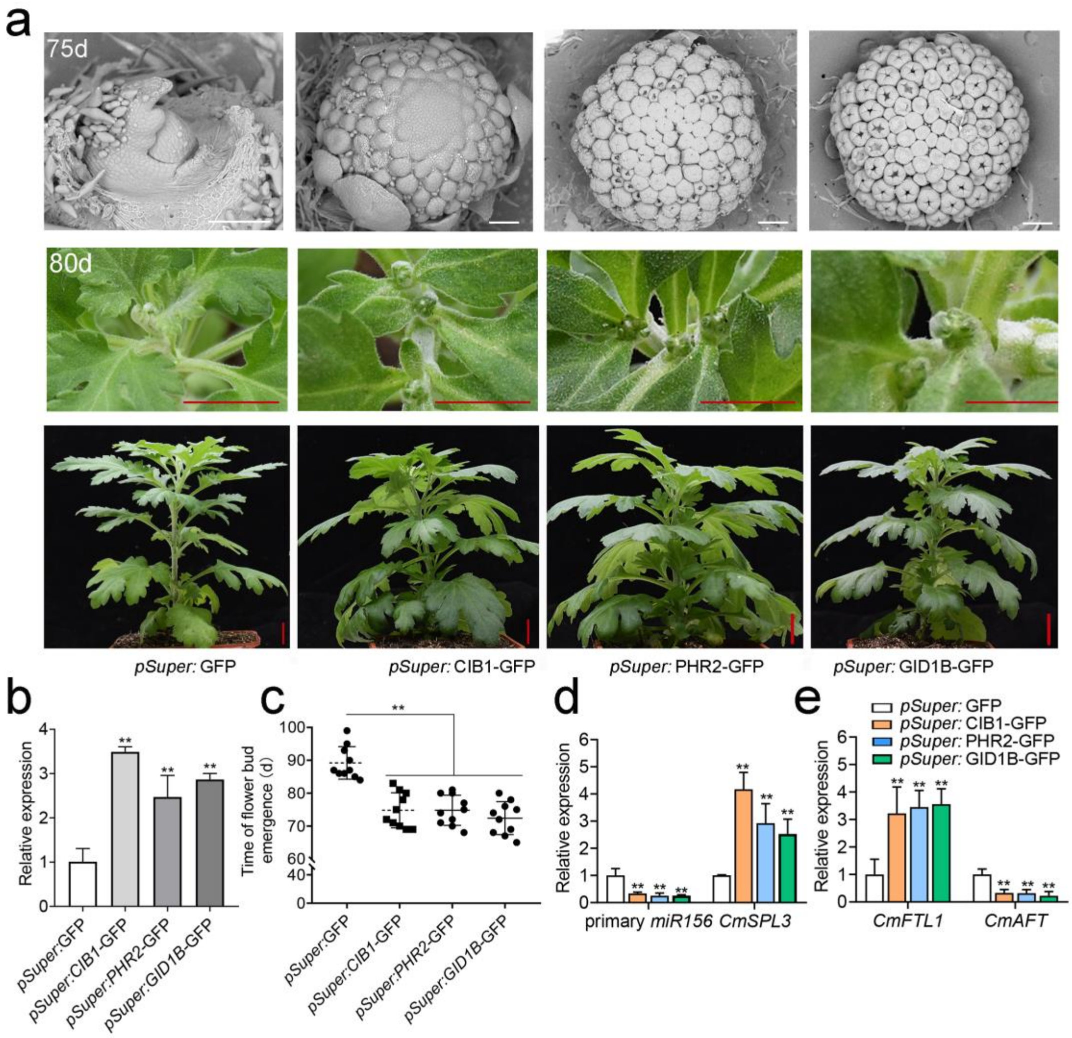

Supplemental Figure S3. Transient overexpression of *CmCIB1*, *CmPHR2* and *CmGID1B* plants flowering early. a. Shoot apices and representative phenotypes of WT plants (50-day-old) infected with *pSuper:*GFP, *pSuper:*CmCIB1-GFP, *pSuper:*CmPHR2-GFP or *pSuper:*CmGID1B-GFP after 20 days under LD conditions and 10 days under SD conditions. White scale bars, 100 um; Red scale bars, 1cm. b. Relative expression levels of *CmCIB1, CmPHR2* or *CmGID1B* analyzed by RT-qPCR in WT plants (50-day-old) infected with *pSuper:*GFP, *pSuper:*CmCIB1-GFP, *pSuper:*CmPHR2-GFP or *pSuper:*CmGID1B-GFP after 5 days under LD conditions. c. Time of flower bud emergence of WT plants (50-day-old) infected with *pSuper:*GFP, *pSuper:*CmCIB1-GFP, *pSuper:*CmPHR2-GFP or *pSuper:*CmGID1B-GFP. d-e. Relative expression levels of primary *cmo-miR156* and *CmSPL3* (d), and *CmFTL1* and

CmAFT (e) analyzed by RT-qPCR in WT plants (50-day-old) infected with *pSuper:*GFP, *pSuper:*CmCIB1-GFP, *pSuper:*CmPHR2-GFP or *pSuper:*CmGID1B-GFP after 20 days under LD conditions and 5 days under SD conditions. The results are the means of three biological replicates with standard deviation in b, d, e. Asterisks indicate significant differences according to a Student's *t*-test in b-e (***P* < 0.01).

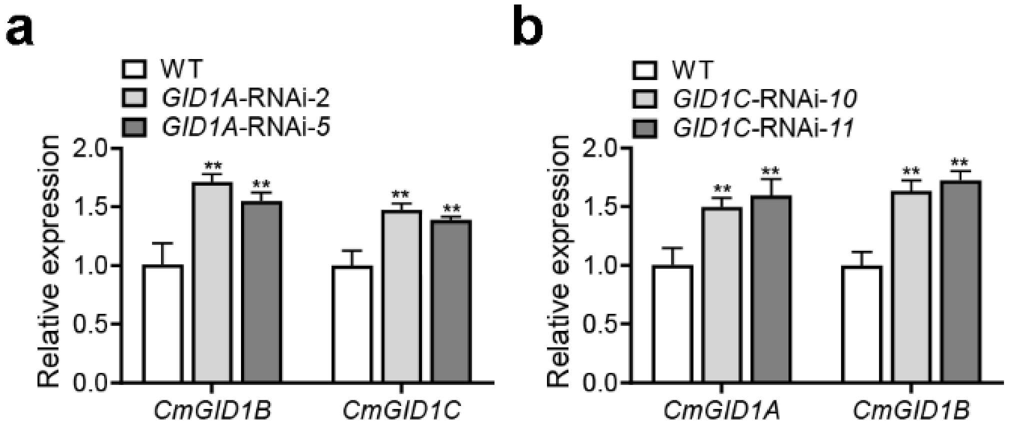

Supplemental Figure S4. Relative expression of the other two *GID1* members in *CmGID1A*-RNAi or *CmGID1C*-RNAi plants. a. Expression of *CmGID1B* and *CmGID1C* was analyzed by RT-qPCR in WT and *CmGID1A*-RNAi plants. b. Expression of *CmGID1A* and *CmGID1B* was analyzed by RT-qPCR in WT and *CmGID1C*-RNAi plants. The results are the means of three biological replicates with standard deviation. Asterisks indicate significant differences according to a Student's *t*-test in a, b (***P* < 0.01).

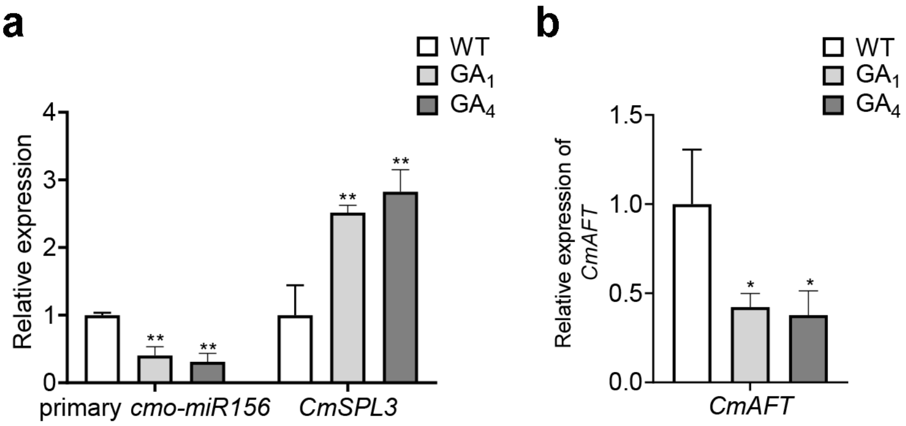

Supplemental Figure S5. Relative expression levels of primary *cmo-miR156*, *CmSPL3*, and *CmAFT* with GA treatment. a-b. Expression of primary *cmo-miR156* and *CmSPL3* (a), and *CmAFT* (b) was analyzed by RT-qPCR in WT plants after GA treatment. The results are the means of three biological replicates with standard deviation. Asterisks indicate significant differences according to a Student's *t*-test in a, b (*$P < 0.05$, **$P < 0.01$).

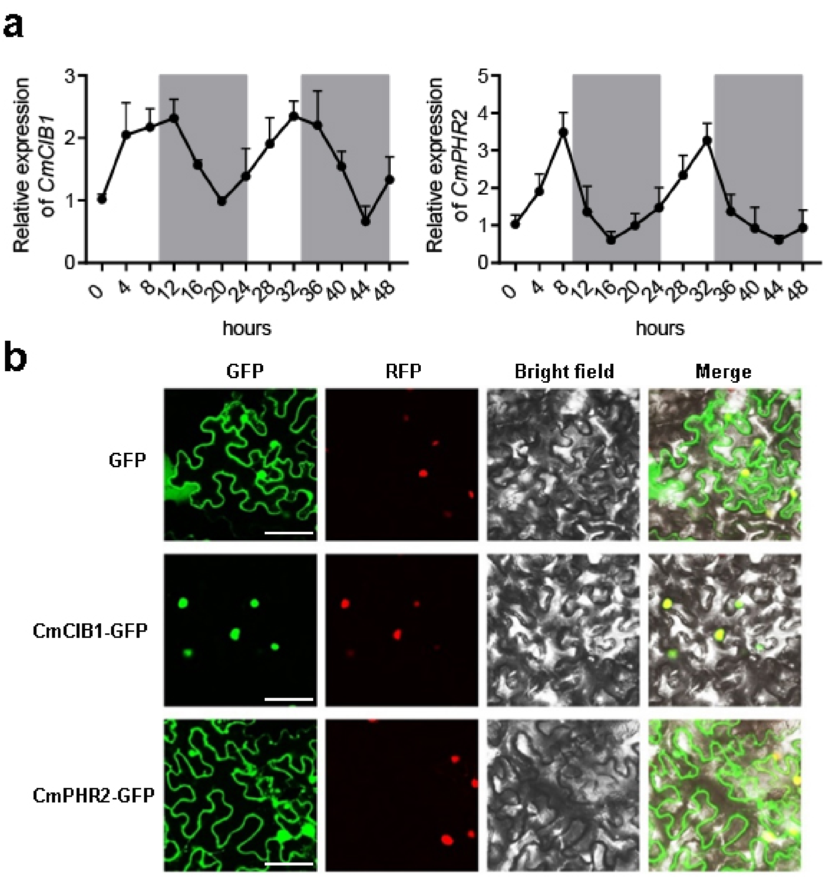

Supplemental Figure S6. Diurnal rhythm analysis of *CmCIB1* and *CmPHR2* expression and subcellular localization analysis of CmCIB1 and CmPHR2. a. Diurnal rhythm analysis of *CmCIB1* and *CmPHR2* expression within 48 hours. The results are the means of three biological replicates with standard deviation. b. Subcellular localization analysis of CmCIB1 and CmPHR2 in the leaves of *N. benthamiana*; empty GFP was used as a control. The green fluorescence is the GFP signal, and the red fluorescence represents the nucleus. Scale bars, 100 μm.

Supplemental Table S1. Primers Used in This Study

Primer Name	Primer Sequence (5'→3')	Construct
Chrysanthemum Transformation		
GID1A-AscI-F	ttacaattaccatggggcgcgcc TAGAGGGTACATCCCCAACC	RNAi-*CmGID1A*
GID1A -SwaI-R	catgttcatctggggatttaaatATGTTATTTGGTTCTGTTGCT	
GID1A -BamHI-F	cgatctctttgatggggatccATGTTATTTGGTTCTGTTGCT	
GID1A -PacI-R	gactctagggactagttaattaaTAGAGGGTACATCCCCAACC	
GID1B-AscI-F	ttacaattaccatggggcgcgccTTTAATAACCCTTTTGTTTATAGG	RNAi-*CmGID1B*
GID1B -SwaI-R	catgttcatctggggatttaaatGAGACACCCTCTTCAAACATCC	
GID1B -BamHI-F	cgatctctttgatggggatccGAGACACCCTCTTCAAACATCC	
GID1B -PacI-R	gactctagggactagttaattaaTTTAATAACCCTTTTGTTTATAGG	
GID1C-AscI-F	ttacaattaccatggggcgcgccGGTTAAGGAGGAAGTGATAC	RNAi-*CmGID1C*
GID1C -SwaI-R	catgttcatctggggatttaaatGTAATAGATCAACTTAGTAGCAC	
GID1C -BamHI-F	cgatctctttgatggggatccGTAATAGATCAACTTAGTAGCAC	
GID1C -PacI-R	gactctagggactagttaattaaGGTTAAGGAGGAAGTGATAC	
GID1BC-AscI-F	ttacaattaccatggggcgcgccGGTTAAGGAGGAAGTGATAC	RNAi-*CmGID1BC*
GID1BC -SwaI-R	catgttcatctggggatttaaatGAGACACCCTCTTCAAACATCC	
GID1BC-BamHI-F	cgatctctttgatggggatccGAGACACCCTCTTCAAACATCC	
GID1BC -PacI-R	gactctagggactagttaattaaGGTTAAGGAGGAAGTGATAC	
CIB1-AscI-F	ttacaattaccatggggcgcgccCTTCAGAAACGCCTAATCCTGC	RNAi-*CmCIB1*
CIB1-SwaI-R	catgttcatctggggatttaaatGGTCCCTGCCAATGTTTCCA	
CIB1-BamHI-F	cgatctctttgatggggatccGGTCCCTGCCAATGTTTCCA	
CIB1-PacI-R	gactctagggactagttaattaaCTTCAGAAACGCCTAATCCTGC	
PHR2-AscI-F	ttacaattaccatggggcgcgccGCGAAACAAAATAATGCTGCTC	RNAi-*PHR2*
PHR2-SwaI-R	catgttcatctggggatttaaatGGCGTCACATGCAGATGCTT	
PHR2-BamHI-F	cgatctctttgatggggatccGGCGTCACATGCAGATGCTT	
PHR2-PacI-R	gactctagggactagttaattaaGCGAAACAAAATAATGCTGCTCC	
Subcellular localization		
1300-GID1A-F	ccaaatcgactctagtctagaATGTCTCAATTCAAGCTAGCTTACA	pCAMBIA1300-GID1A
1300-GID1A-R	gcccttgctcaccatggtaccCTATTCACATTCGGATGTTGG	
1300-GID1B-F	ccaaatcgactctagtctagaATGCTCAGGCGCCCAGATGG	pCAMBIA1300-GID1B
1300-GID1B-R	gcccttgctcaccatggtaccTCAAGATCTCACAAAGTTTTCGAT	
1300-GID1C-F	ccaaatcgactctagtctagaATGCTCCGGCGACCGGACGG	pCAMBIA1300-GID1C
1300-GID1C-R	gcccttgctcaccatggtaccATGCTCCGGCGACCGGACGG	
1300-CIB1-F	ccaaatcgactctagtctagaATGAGTCACGAATTGCGAAA	pCAMBIA-1300-CIB1
1300-CIB1-R	gcccttgctcaccatggtaccTCACATTTCCATCTTCAAAT	
1300-PHR2-F	ccaaatcgactctagtctagaATGGAATCATCATCAACAAA	pCAMBI

1300-PHR2-R	gcccttgctcaccatggtaccTCATGCGGCTGCACCTGTAC	A-1300-PHR2
Yeast assay		
proGID1B-KpnI-F-1	cttgaattcgagctcggtaccTCTCCCACGATTATCTACCGTCG	pAbAi-proGID1B
proGID1B-XhoI-R-1	atacagagcacatgcctcgagTTTTTTCTTAGTGTTGATGTTTAGT	
proGID1B-KpnI-F-2	cttgaattcgagctcggtaccTGGATTGAGGGAGTACACAC	
proGID1B-XhoI-R-2	atacagagcacatgcctcgagCATTTTTAGACTATCTCACCTC	
proGID1B-KpnI-F-3	cttgaattcgagctcggtaccTTTTTTACTATTATTATTAAAATG	
proGID1B-XhoI-R-3	atacagagcacatgcctcgagCAAGATTCAAGAAAACAAAAC	
CIB1-EcoRI-F	gccatggaggccagtgaattcATGAGTCACGAATTGCGAA	pGADT7-CIB1
CIB1-BamHI-R	cagctcgagctcgatggatccTCACATTTCCATCTTCAAATTGC	
PHR2-EcoRI-F	gccatggaggccagtgaattcATGGAATCATCATCAACAAAAA	pGADT7-PHR2
PHR2-BamHI-R	cagctcgagctcgatggatccTCATGCGGCTGCACCTGTAC	
PHR2-EcoRI-F	atggccatggaggccgaattcATGGAATCATCATCAACAAA	pGBKT7-PHR2
PHR2-SalI-R	atgcggccgctgcaggtcgacTCATGCGGCTGCACCTGT	
GUS-EcoRI-F	gccatggaggccagtgaattcATGTTACGTCCTGTAGAAAC	pGADT7-GUS
GUS-BamHI-R	cagctcgagctcgatggatccTCATTGTTTGCCTCCCTGCT	
GUS-EcoRI-F	atggccatggaggccgaattcATGTTACGTCCTGTAGAAAC	pGBKT7-GUS
GUS-SalI-R	atgcggccgctgcaggtcgacTCATTGTTTGCCTCCCTGCT	
Dual-Luciferase Reporter Assay		
ProGID1B- HindIII-F	gtcgacggtatcgataagcttTGGATTGAGGGAGTACACACTACC	pGreenII-LUC-ProGID1B
ProGID1B- BamHI-R	cgctctagaactagtggatccCAAGATTCAAGAAAACAAAAC	
CIB1-EcoRI-F	tcccccgggctgcaggaattcATGAGTCACGAATTGCGAAATG	pGreenII-SK-CIB1
CIB1-KpnI-R	tgatttcagcgaattggtaccTCACATTTCCATCTTCAAATTGCT	
PHR2-EcoRI-F	tcccccgggctgcaggaattcATGGAATCATCATCAACAAA	PGreenII-SK-PHR2
PHR2-KpnI-R	tgatttcagcgaattggtaccCATGCGGCTGCACCTGTAC	
ChIP Assay		
CIB1- XbaI-F	ccaaatcgactctagtctagaATGAGTCACGAATTGCGAAATG	pSuper1300 (GFP-C)-CIB1
CIB1-KpnI-R	gcccttgctcaccatggtaccCATTTCCATCTTCAAATTGCTTCC	
PHR2-XbaI-F	ccaaatcgactctagtctagaATGGAATCATCATCAACAAA	pSuper1300 (GFP-C)-PHR2
PHR2-KpnI-R	gcccttgctcaccatggtaccTGCGGCTGCACCTGTACAA	
ProGID1B-Chip-F1	TGGATTGAGGGAGTACACACTACC	
ProGID1B-Chip-R1	GTGTGGCTAAAAATACTTCTAACCTTTC	
ProGID1B-Chip-F2	GAAAGGTTAGAAGTATTTTTAGCCACAC	
ProGID1B-Chip-R2	AGTCTTTCTGTGCAGGTGGT	
ProGID1B-Chip-F3	ATAGTCACCACCACCTGCAC	
ProGID1B-Chip-R3	TGCCTTTTCTTCTTACCATTCATT	
ProGID1B-Chip-F4	TTGGCACTAATGCCCTGTCG	
ProGID1B-Chip-R4	TAGCCATAGCCTGACCCGAA	

ProGID1B-Chip-F5	CAGCTTCGGGTCAGGCTATG	
ProGID1B-Chip-R5	TTGGCTAACTATGGTTCGGGT	
ProGID1B-Chip-F6	CAATCAGGTTGACCCGAACCA	
ProGID1B-Chip-R6	AGGAAGTGTGGTAGAGCGAA	
ProGID1B-Chip-F7	TCCTTCGCTCTACCACACTTCC	
ProGID1B-Chip-R7	CCTTAAAAGAGTATTTGACTAGGCATG	
BIFC		
CIB1- XbaI-F	gagaacacgggggactctagaATGAGTCACGAATTGCGAAATG	35S-SPYCE(M)-CIB1
CIB1-KpnI-R	gtacatcccgggagcggtaccCATTTCCATCTTCAAATTGCTTCC	
PHR2-XbaI-F	gagaacacgggggactctagaATGGAATCATCATCAACAAA	35S-SPYNE(R)173-PHR2
PHR2-KpnI-R	ctccatcccgggagcggtaccTGCGGCTGCACCTGTACAA	
In situ Hybridization		
HAN-Sp6-GID1B	GATTTAGGTGACACTATAGaatGCTATGGCTGGCAGTAATGAAAT	GID1B-probe
HAN-T7-GID1B	tgTAATACGACTCACTATAGGGAGATCTCACAAAGTTTTCGA	
HAN-Sp6-CIB1	GATTTAGGTGACACTATAGaatGCTATGAGTCACGAATTGCGAAATGACGGCCCG	CIB1-probe
HAN-T7-CIB1	tgTAATACGACTCACTATAGGGCATTTCCATCTTCAAATTGCTTCCTTC	
HAN-Sp6-PHR2	GATTTAGGTGACACTATAGaatGCTATGGAATCATCATCAACAAAAAAACCAC	PHR2-probe
HAN-T7-PHR2	tgTAATACGACTCACTATAGGGTGCGGCTGCACCTGTACAAGCTGTAACTGG	
Pull-down assays		
CIB1-BamHI-F	cagcaaatgggtcgcggatccATGAGTCACGAATTGCGAAA	pET-28a-CIB1
CIB1-EcoRI-R	ttgtcgacggagctcgaattcTCACATTTCCATCTTCAAATT	
PHR2-BamHI-F	gatctggttccgcgtggatccATGGAATCATCATCAACAAA	pGEX-4T-2-PHR2
PHR2-EcoRI-R	ctcgagtcgacccgggaattcCATGCGGCTGCACCTGTAC	
RT-qPCR		
GID1A-F	TAATTTCATGTGCCACCGCC	
GID1A-R	TCCTGGCGTCCTCTCATTTG	
GID1B-F	TGGGATATCTCTCGGGCAGG	
GID1B-R	GAGACACCCTCTTCAAACATCCA	
GID1C-F	TCTACTTGTGGGATTATGAGAGGA	
GID1C-R	CCATAAACAATCATCAAAAACCCCT	
GA20ox1-F	TCACTCAGAAGCATTACAGGGC	
GA20ox1-R	GCATCCTCTTTGGTGGTCATCT	
GA3ox1-F	AAGCTGAGGATAGCTTTGTATGA	
GA3ox1-R	TGTGTCGGATTGTATGTCGTCT	

FTL1-F	AATCGTGTGCTATGAGAGCC	
FTL1-R	GCTTGTAACGTCCTCTTCATGC	
SPL3-F	AGCCATGGAGGCCACTTTTT	
SPL3-R	GTGGACGTCAACAGCGTTTC	
Pri-cmo-MIR156-F	AAAGAGGAAAGCTGCACATTAGG	
Pri-cmo-MIR156-R	GCTTCAAGCATATCAAACTATTTGC	
In situ Hybridization		
HAN-Sp6-GIDB	GATTTAGGTGACACTATAGaatGCTATGGCTGGCAGTAATGAAAT	
HAN-T7-GIDB	tgTAATACGACTCACTATAGGGAGATCTCACAAAGTTTTCGA	
HAN-Sp6-CIB1	GATTTAGGTGACACTATAGaatGCTATGAGTCACGAATTGCGAAATGACGGCCCG	
HAN-T7- CIB1	tgTAATACGACTCACTATAGGGCATTTCCATCTTCAAATTGCTTCCTTC	
HAN-Sp6-PHR2	GATTTAGGTGACACTATAGaatGCTATGGAATCATCATCAACAAAAAAACCAC	
HAN-T7- PHR2	tgTAATACGACTCACTATAGGGTGCGGCTGCACCTGTACAAGCTGTAACTGG	
